# Supplementary material for: Local gate control of Mott metal-insulator transition in a 2D metal-organic framework
Source: Nat Commun. 2024 Apr 26;15:3559. doi: 10.1038/s41467-024-47766-8 (PMC11053079; doi:10.1038/s41467-024-47766-8)
Supplement: Supplementary file 1 — Supplementary Information [file 41467_2024_47766_MOESM1_ESM.pdf]

Supplementary Information: Local gate control of Mott metal-insulator transition in a 2D metal-organic framework

Benjamin Lowe[1,2†], Bernard Field[1,2†], Jack Hellerstedt[1,2], Julian Ceddia[1,2], Henry L. Nourse[3], Ben J. Powell[4*], Nikhil V. Medhekar[2,5*] and Agustin Schiffrin[1,2*]

[1]School of Physics and Astronomy, Monash University, Clayton, Victoria 3800, Australia.
[2]ARC Centre of Excellence in Future Low-Energy Electronics Technologies, Monash University, Clayton, Victoria 3800, Australia.
[3]Quantum Information Science and Technology Unit, Okinawa Institute of Science and Technology Graduate University, Onna-son, Okinawa 904-0495, Japan.
[4]School of Mathematics and Physics, The University of Queensland, Brisbane, Queensland 4072, Australia.
[5]Department of Materials Science and Engineering, Monash University, Clayton, Victoria 3800, Australia.

*Corresponding author(s). E-mail(s): powell@physics.uq.edu.au; nikhil.medhekar@monash.edu; agustin.schiffrin@monash.edu;
†These authors contributed equally to this work.

Contents

Supplementary Note 1 **DFT calculations: large energy-window band structure** **3**

Supplementary Note 2 **Dynamical mean-field theory calculations** **3**

Supplementary Note 3 Absence of quasiparticle peaks in metallic regime 7

Supplementary Note 4 DMFT calculations: spectral functions $A(E)$ for different electron fillings 9

Supplementary Note 5 DMFT calculations: k-resolved spectral function 10

Supplementary Note 6 Growth and commensurability 11

Supplementary Note 7 Experimental dI/dV vs. DMFT spectral functions 14

Supplementary Note 8 Bias-dependent STM imaging 17

Supplementary Note 9 dI/dV maps 18

Supplementary Note 10 STS measurements at MOF high-symmetry points 20

Supplementary Note 11 Electronic influence of defects 21

Supplementary Note 12 Broad energy-range STS measurements 24

Supplementary Note 13 Determination of band edges 25

Supplementary Note 14 Moiré-dependent STS measurements at DCA lobe sites of DCA_3Cu_2 MOF 26

Supplementary Note 15 STS measurements for hBN/Cu(111) moiré domains with different periods 29

Supplementary Note 16 Charging signatures 33

Supplementary Note 17 Charging features in MOF dI/dV at pore-wire boundary of moiré pattern 34

Supplementary Note 18 Estimate of tip work function 36

Supplementary Note 19 DBTJ model: energy diagrams 38

Supplementary Note 20 Tip-induced gating at moiré pore region 41

Supplementary Note 21 Tip-induced gating at Cu sites; DBTJ model fit parameters 43

Supplementary Note 22 Metallic Character 48

Supplementary Note 23 Temperature-dependent measurements and calculations 49

Supplementary Note 24 STS measurements at different moiré pore regions 50

Supplementary Note 1 DFT calculations: large energy-window band structure

In the main text, we showed DFT calculations of the DCA_3Cu_2/hBN/Cu(111) band structure over an energy window from -0.5 to 0.5 eV (Fig. 1d). Supplementary Fig. 1 shows the same calculated band structure over a wider energy window from -3 to 3 eV, for both the freestanding MOF (Supplementary Fig. 1a) and the MOF on hBN/Cu(111) (Supplementary Fig. 1b). These calculations show that the three near-Fermi MOF kagome bands are well isolated and separated in energy by more than 1 eV from the other bands. This is why we focus on these near-Fermi electronic states throughout the main text.

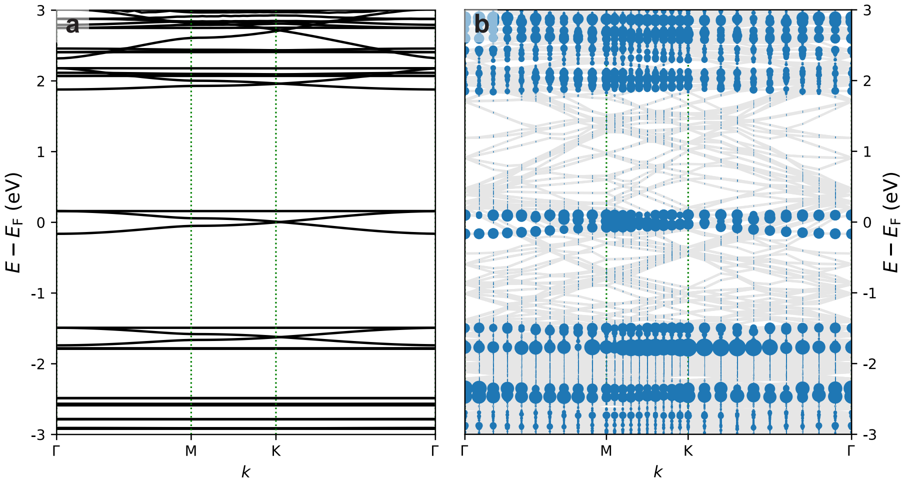

Supplementary Fig. 1: **DFT band structures of DCA_3Cu_2 MOF over wide energy range. a,** Freestanding MOF. **b,** MOF on hBN/Cu(111). Grey curves: Cu(111) and hBN bands. Blue circles: projections onto MOF states.

Supplementary Note 2 Dynamical mean-field theory calculations

We performed dynamical mean-field theory (DMFT) calculations for the freestanding kagome lattice (see Methods in main text), for different values of

Hubbard on-site Coulomb repulsion U and chemical potential E_{F}. The chemical potential is referenced with respect to the Dirac point of the free-standing kagome lattice density of states (DOS) (i.e., the $U = 0$ spectral function $A(E)$). We used a nearest-neighbour hopping energy $t = 0.05$ eV, matching the DFT-calculated band structure [1]. We calculated $A(E)$ for different values of U at half-filling (i.e., one electron per site, with $E_{\mathrm{F}} \approx 3t/2 + U/2$), and for different values of E_{F} at a fixed $U = 0.65$ eV, see Supplementary Fig. 2.

Supplementary Fig. 2a shows a Mott insulating phase emerging at half-filling for all $U \geq U_{\mathrm{crit}} \approx 0.55$ eV. The energy gap increases with increasing U (see dashed lines in Supplementary Fig. 2a). For $U < U_{\mathrm{crit}}$, the on-site Coulomb repulsion is not sufficiently large (relative to the bandwidth 0.3 eV of the non-interacting kagome band structure) to open a Mott gap; the spectral functions $A(E)$ resemble the non-interacting (i.e., single-electron) DOS.

Supplementary Fig. 2b displays the variation of $A(E)$ with E_{F}, at a fixed $U = 0.65$ eV. At this value of U, the bandgap and Mott insulating phase persist across a chemical potential window of $\sim$0.25 eV ($5t \leq E_{\mathrm{F}} \leq 9.5t$). When E_{F} increases (decreases), these bands progressively shift to lower (higher, respectively) energy. Outside this range of E_{F}, the Mott phase breaks down and $A(E)$ starts resembling again the non-interacting DOS given by the tight-binding model (when $U = 0$).

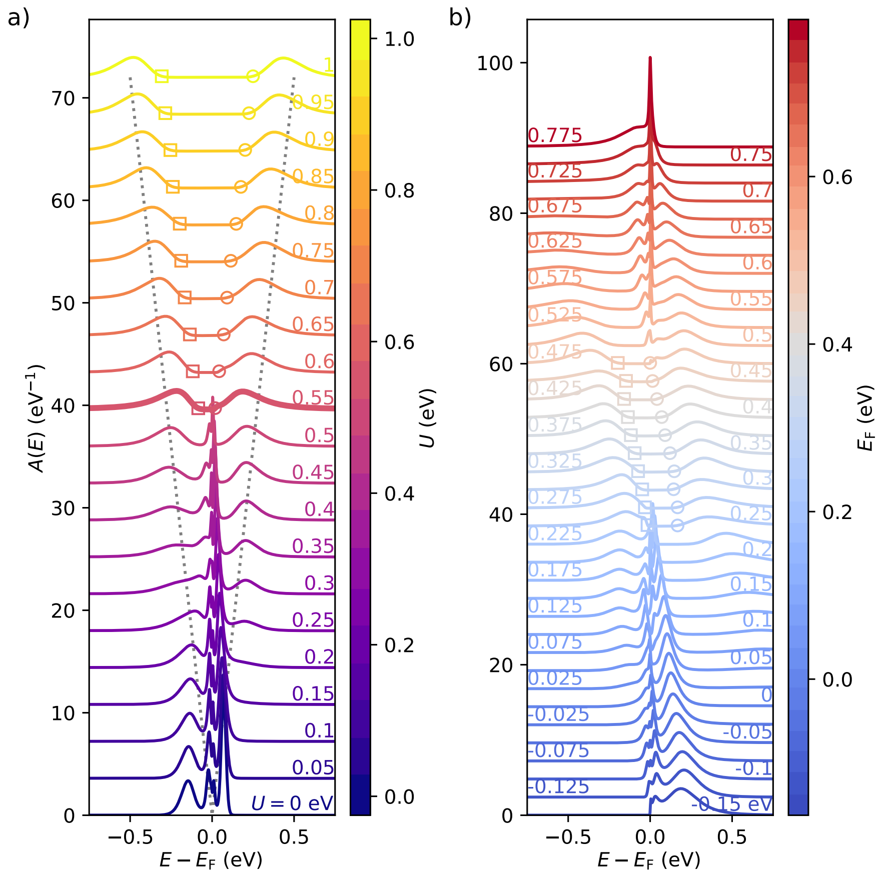

Supplementary Fig. 2: **Spectral functions $A(E)$ calculated by DMFT for the free-standing kagome lattice. a,** Spectra for different on-site Coulomb repulsion energies, U, with chemical potential, E_{F} adjusted to maintain half-filling of the kagome three-band band structure ($E_{\mathrm{F}} \approx 1.5t + U/2$; $t = 0.05$ eV). The spectra for $U < U_{\mathrm{crit}}$, where $U_{\mathrm{crit}} \approx 0.55$ eV (bold spectrum), correspond to the trivial metallic phase. For $U > U_{\mathrm{crit}}$, the spectra reveal a correlated-electron Mott insulating phase. Grey dashed lines mark $\pm U/2$, where the Hubbard bands are expected to be observed. Squares (circles): lower Hubbard band maxima, LHBM (upper Hubbard band minima, UHBM, respectively). **b,** Spectral functions for different values of E_{F}, at fixed $U = 0.65$ eV. Spurious dips at $E = E_{\mathrm{F}}$ and additional broadening away from E_{F} are due to limitations of analytic continuation approach [2].

The spectral functions $A(E)$ in Supplementary Fig. 2b correspond to those in Fig. 3d of the main text. In Fig. 3c, d of the main text, we determined the correspondence between experimental $\mathrm{d}I/\mathrm{d}V$ spectra – acquired at different

positions x along two pore regions of the hBN/Cu(111) moiré domain in Fig. 3a of the main text – and theoretical $A(E)$ curves (each calculated for a uniform system and characterised by a specific chemical potential E_{F}), by assuming a sinusoidal variation of the experimental local work function, $\Delta\Phi$, as a function of x. For a periodicity $\lambda \approx 12.5$ nm for this specific moiré domain, $0.15 \lesssim \Delta\Phi \lesssim 0.3$ eV based on experimental estimates [3]. In our calculations, we took account of this experimental $\Delta\Phi(x)$ by varying the chemical potential E_{F} of the uniform model kagome system accordingly. A chemical potential variation amplitude $\Delta E_{\mathrm{F}} = 0.2$ eV (Fig. 3c of main text) yielded, for the DMFT-calculated $A(E)$ curves, lower Hubbard band maxima and upper Hubbard band minima that matched simultaneously those of our experimental $\mathrm{d}I/\mathrm{d}V$ results (Fig. 3e of main text).

Supplementary Note 3 Absence of quasiparticle peaks in metallic regime

Some of the DMFT-calculated spectral functions $A(E)$ in Fig. 3d of the main text and Supplementary Fig. 2 show sharp peaks near $E = E_{\mathrm{F}}$ in the metallic regime. We attribute these peaks to Landau Fermi-liquid quasiparticles [4]. These peaks are not observed in the experimental $\mathrm{d}I/\mathrm{d}V$ spectra in Fig. 3b of the main text. In our DMFT calculations the peaks correspond to quasiparticles with a mean free path $\ell \gtrsim 10$ nm. This is of the order of the period of the hBN/Cu(111) moiré domain, and is much larger than the width of the hBN/Cu(111) moiré wire regions (∼4 nm). Hence, since quasiparticles can only propagate coherently over the finite width of the wire regions and are strongly scattered by the pore regions, the quasiparticle peak is suppressed.

In what follows we rationalise our estimate of $\ell \gtrsim 10$ nm. The mean-free path of an electron near the Fermi surface is given by $\ell_{\mathbf{k}} = -\hbar v(\mathbf{k})/(2\mathrm{Im}[\Sigma(\mathbf{k}, E)])$ [5], where $v(\mathbf{k})$ is the magnitude of the non-interacting group velocity, and $\Sigma(\mathbf{k}, E)$ is the many-body self-energy. In a Fermi liquid, as $T \to 0$, $\mathrm{Im}[\Sigma(\mathbf{k}, E)] \to 0$ at the Fermi surface ($E \to E_{\mathrm{F}}$ and $\mathbf{k} \to \mathbf{k}_{\mathrm{F}}$) and the mean-free path is infinite. In DMFT, $\Sigma(\mathbf{k}, E)$ is approximated to have no dependence on $\mathbf{k}$; we can hence make the replacement $\Sigma(\mathbf{k}, E) \to \Sigma(E)$.

For the two bottom Dirac bands of the band structure of the non-interacting kagome system, the magnitude of the group velocity is

$$v(\mathbf{k}) = \frac{at}{\hbar}\sqrt{\frac{\sin^2\left(\frac{ak_x}{2}\right)\left(2\cos\left(\frac{ak_x}{2}\right)+\cos\left(\frac{\sqrt{3}ak_y}{2}\right)\right)^2 + 3\cos^2\left(\frac{ak_x}{2}\right)\sin^2\left(\frac{\sqrt{3}ak_y}{2}\right)}{1+4\cos^2(ak_x/2)+4\cos(ak_x/2)\cos(\sqrt{3}ak_y/2)}}. \tag{1}$$

where a is the kagome lattice constant. The non-interacting Fermi surface is given by the wavevectors satisfying

$$k_y = \pm\frac{2}{\sqrt{3}a}\arccos\left(\frac{(E_{\mathrm{F}}/2t)^2 - 1/4 - \cos^2(ak_x/2)}{\cos(ak_x/2)}\right). \tag{2}$$

For a value of E_F corresponding to an electron occupation [6] of $n = 0.915$ in the middle of the wire region (n taken from the calculation in Supplementary Fig. 3, with E_F differing here due to using $U = 0$ for calculating v_{F}; $n = 2$ corresponds to fully occupied kagome bands), using $a = 2.0432$ nm, the average non-interacting Fermi velocity is $v_{\mathrm{F}} = (1.3\pm0.3)\times10^5$ ms^{-1}, where ± indicates the range of values $v(\mathbf{k})$ takes at $E = E_{\mathrm{F}}$. Note that at $n = 1$ (half-filling), $v_{\mathrm{F}} = 1.4 \times 10^5$ ms^{-1}.

In our DMFT calculations, $\Sigma(E)$ is computed as a function of discrete Matsubara frequencies [5], $\omega_n = (2n+1)\pi k_{\mathrm{B}}T/\hbar$, where n is an integer, k_{B} is the Boltzmann constant, and T is the temperature. In order to calculate $\Sigma(E)$ (for arbitrary energy E), we can perform analytic continuation [2] to real frequencies, $\omega = E/\hbar$. This procedure is computationally costly due to noise

in numerical quantum Monte Carlo calculations. However, $\Sigma(E = E_\mathrm{F} \pm \pi k_\mathrm{B} T)$ can be obtained from the lowest Matsubara frequency by the straightforward analytic continuation, $i\omega_n \to \omega - E_\mathrm{F}\hbar + i\eta$, where η is an infinitesimal real number. Since $\mathrm{Im}[\Sigma(i\omega_n)]$ is a monotonic function in the vicinity of $\omega_{n=0}$, $|\mathrm{Im}[\Sigma(i\omega_{n=0})]|$ is an overestimate of $|\mathrm{Im}[\Sigma(E = E_\mathrm{F})]|$ by analytic continuation. Thus, $\mathrm{Im}[\Sigma(i\omega_{n=0})]$ provides an underestimate of the mean-free path of a typical quasiparticle, as statistically most occupied states have an energy $\omega < \omega_{n=0}$. Therefore, the mean-free path of a typical quasiparticle can be estimated as

$$\ell \gtrsim -\frac{\hbar v_\mathrm{F}}{2\mathrm{Im}[\Sigma(i\omega_{n=0})]}. \tag{3}$$

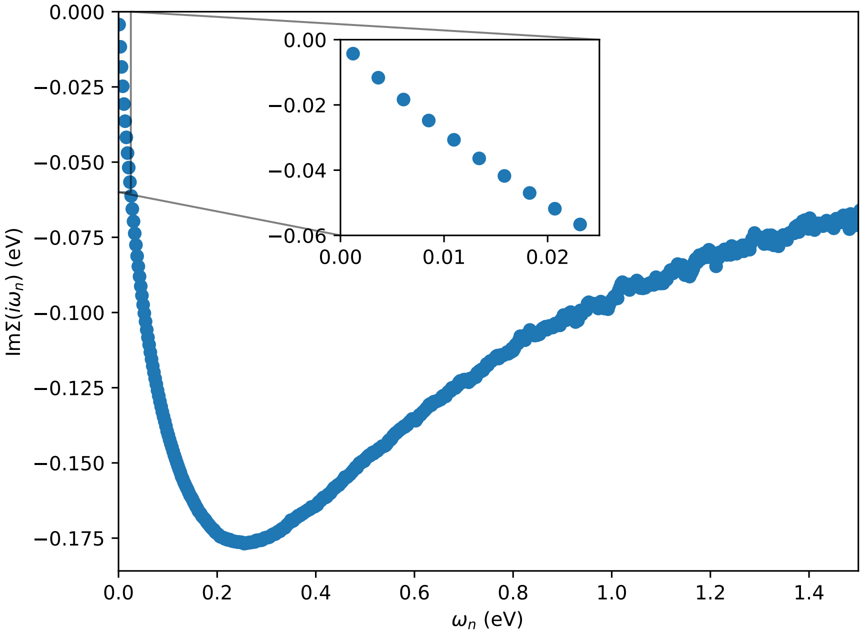

Supplementary Fig. 3: Imaginary part of the self-energy $\Sigma(i\omega_n)$ as a function of Matsubara frequencies ω_n, calculated by DMFT for $U = 0.65$ eV, $E_\mathrm{F} = 0.2$ eV and $T = 4.5$ K.

Figure 3d of the main text shows spectral functions $A(E)$ calculated via DMFT, with $U = 0.65$ eV, for different chemical potentials E_F (relative to the Dirac point of the kagome band structure in the non-interacting case). In particular, for $E_\mathrm{F} = 0.2$ eV, the system is in a metallic regime, away from half-filling and from the Mott insulating regime, similar to the experimental measurements performed at the wire region of the hBN/Cu(111) moiré pattern (see Supplementary Fig. 2b). Note that in this metallic regime, our DMFT

calculations of $\Sigma(i\omega_n)$ converge by only considering low Matsubara frequencies ω_n; this facilitates calculations at low temperatures [7]. Supplementary Fig. 3 shows the imaginary part of the DMFT-calculated self-energy $\Sigma(i\omega_n)$ as a function of ω_n. We find that $\mathrm{Im}[\Sigma(i\omega_{n=0})] \approx -0.00429$ eV, giving a mean-free path for a typical quasiparticle of $\ell \gtrsim 10$ nm.

Supplementary Note 4 DMFT calculations: spectral functions $A(E)$ for different electron fillings

In Fig. 3 of the main text, the DCA_3Cu_2 MOF is metallic at the wire regions of the hBN/Cu(111) moiré pattern, but with an energy gap at the Fermi level elsewhere. In Fig. 4 of the main text, however, we show that for large tip-sample distances the MOF also appears to be in the Mott insulating regime at the wire region. This difference is related to the different tip-sample distances used in these two figures.

In Fig. 3 of the main text, we used a tip-sample distance, $\Delta z + z_0$, where z_0 is defined by the STM setpoint of $V_\mathrm{b} = 10$ mV, $I_\mathrm{t} = 10$ pA, and $\Delta z = 190$ pm. The largest tip-sample distance used in Fig. 4 of the main text, however, consists of $\Delta z = 330$ pm, with z_0 defined by the same setpoint. Importantly, Fig. 4e of the main text shows that, at the wire region, the MOF is in a gapless metallic phase for a Δz range between 175 and 275 pm; the tip-sample distance used in Fig. 3 of the main text is within this range. Based on Fig. 4e of the main text (which captures the energy shifts of $\mathrm{d}I/\mathrm{d}V$ features with changing Δz), we estimate that the chemical potential E_F could shift by $\sim$45 meV from $\Delta z = 330$ pm to $\Delta z = 190$ pm.

Figure 3d of the main text shows the DMFT-calculated spectral functions, $A(E)$, with a chemical potential E_F modulated by 0.2 eV, to match the expected work function modulation resulting from the hBN/Cu(111) moiré pattern. The agreement between the calculated spectral functions and the experimental $\mathrm{d}I/\mathrm{d}V$ spectra in Fig. 3b provides compelling evidence that the theory captures the fundamental physics observed experimentally. To capture the potential effect of the tip-sample distance difference, we performed the same DMFT calculations of $A(E)$ as in Fig. 3d of the main text, but with E_F shifted upwards by 45 meV (Supplementary Fig. 4). Compared to Fig. 3d of the main text, these spectral functions are all gapped at the Fermi level and in the Mott insulating regime, even for the smallest E_F. This is consistent with the experimental spectra acquired at the wire region with large tip-sample distances (Fig. 4d of the main text), and further corroborates our theoretical model of the Mott insulating DCA_3Cu_2 MOF.

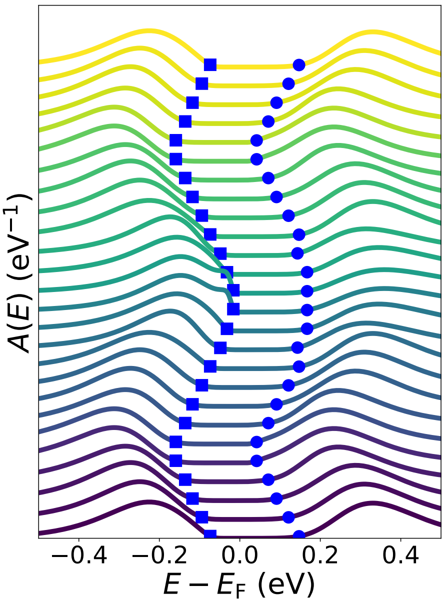

Supplementary Fig. 4: Spectral functions $A(E)$ calculated by DMFT, with 200 meV modulation of the chemical potential, E_F, to match the experimental hBN/Cu(111) moiré work function modulation. This calculation is the same as in Fig. 3 of the main text, but with E_F shifted upwards by 45 meV for all spectra to capture the influence of different tip-sample distances and of tip-induced gating. In this case, all spectra remain gapped at the Fermi level.

Supplementary Note 5 DMFT calculations: k-resolved spectral function

To further investigate the correlated-electron Mott insulating phase of the DCA_3Cu_2 MOF, we calculated via DMFT the k-dependent spectral function, $A(E,\mathbf{k}) = \mathrm{Im}\left[-1/\left[\pi(E+E_\mathrm{F}-\varepsilon(\mathbf{k})-\Sigma(E))\right]\right]$, for $U = 0.65$ eV and $E_\mathrm{F} = 0.4$ eV (corresponding to ∼half-filling of the three near-Fermi MOF kagome bands), where $\varepsilon(\mathbf{k})$ is the non-interacting kagome band structure and $\Sigma(E)$ is the self-energy analytically continued to real energy (Supplementary Fig. 5).

We observe a significant gap at the Fermi level ($E = 0$), with an occupied lower Hubbard band (LHB) with weakly dispersive features (in particular close to the Γ point), and an empty upper Hubbard band (UHB) with significantly less dispersion. The *diffuse* nature of these bands (as opposed to sharp and well-defined, as in conventional band theory) is indicative of band incoherence; this is expected for a Mott insulating phase. The integration of $A(E,\mathbf{k})$ over $\mathbf{k}$ results in the spectral function $A(E)$ shown and discussed throughout the manuscript (e.g., orange curve in Fig. 1e). It is important to

note that DMFT can depict correlated-electron phenomena (including Mott metal-insulator transitions) more reliably and accurately than DFT [8–10].

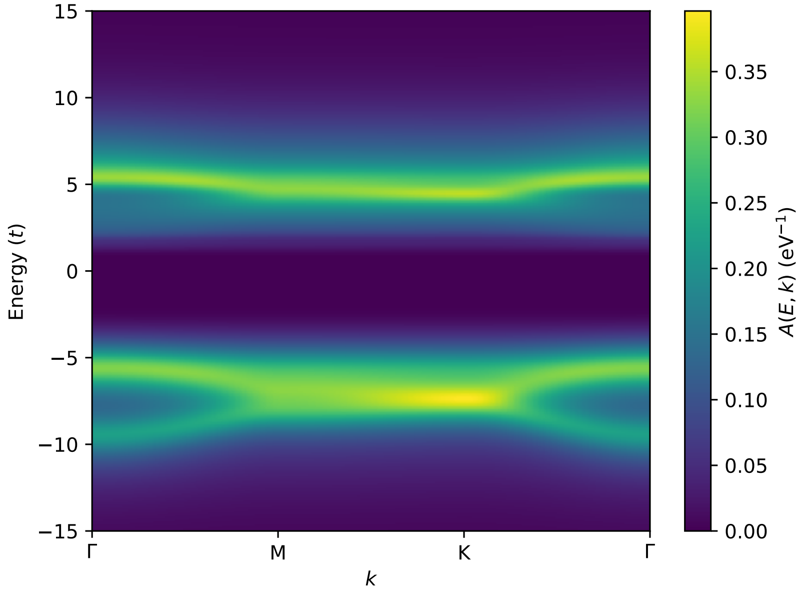

Supplementary Fig. 5: k-resolved spectral function for freestanding DCA_3Cu_2 MOF, calculated by DMFT ($U = 0.65$ eV, $t = 0.05$ eV, $E_F = 0.4$ eV, corresponding to half-filling of the near-Fermi kagome bands).

Supplementary Note 6 Growth and commensurability

In initial preparation attempts of DCA_3Cu_2 on hBN/Cu(111), we performed subsequent depositions of DCA molecules and then Cu atoms, as reported in the Methods section of the main text, but with the substrate held at room temperature (RT) throughout. Characterisation of these surfaces revealed supramolecular arrays of purely DCA molecules (that is, without DCA-Cu coordination) as the dominant phase, consistent with previous findings [11], with only small regions of MOF (Supplementary Fig. 6a-b). We found that, by first depositing DCA with the substrate held at RT, and subsequently depositing Cu with the substrate cooled to ∼77 K, a greater coverage of MOF could be achieved. This low temperature method, however, also stabilised other DCA-Cu coordination motifs, leading to other amorphous structures, e.g., see Supplementary Fig. 6c, d. Further subsequent annealing of the sample

to ~200 K led to monocrystalline DCA_3Cu_2 MOF domains as the most abundant phase, with only small regions of the supramolecular DCA-only phase (Supplementary Fig. 6e). There are some defects (cracks, vacancies, Cu clusters) within the MOF domain (see Supplementary Note 11 for discussion on effects of such defects on the MOF electronic properties).

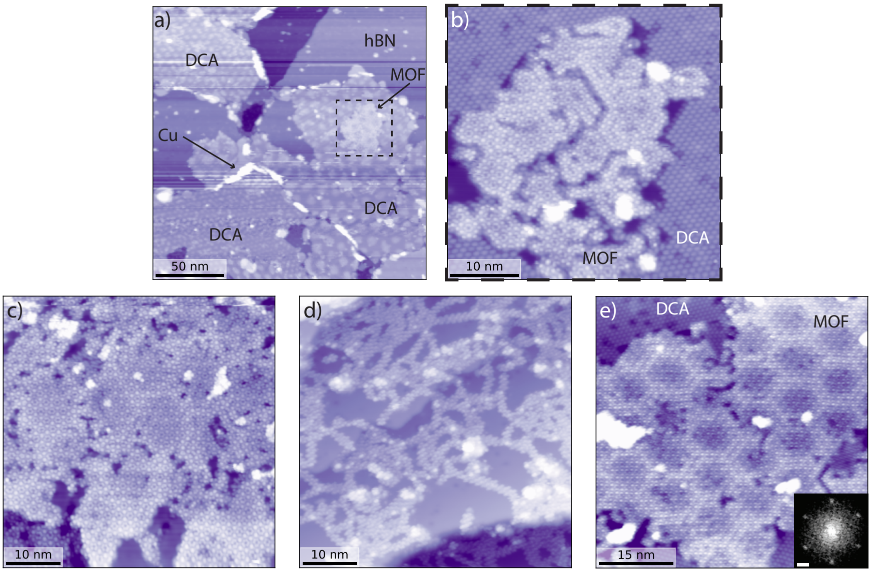

Supplementary Fig. 6: **Growth of DCA_3Cu_2 MOF on hBN/Cu(111). a,** Constant-current STM image showing mostly supramolecular DCA-only domains [11], with one small region of DCA_3Cu_2 MOF (dashed box), after sequential deposition of DCA then Cu with the substrate held at RT (V_b = 1 V, I_t = 10 pA). **b,** Smaller scale constant-current STM image showing the region of MOF highlighted by dashed box in (a) (V_b = -1 V, I_t = 10 pA). **c,** Constant-current STM image showing larger regions of DCA_3Cu_2 MOF after sequential deposition of DCA with the sample at RT and then Cu with the sample at ~77 K (V_b = -1 V, I_t = 10 pA). **d,** Constant-current STM image showing amorphous structures DCA-Cu coordination structures stabilized by the same preparation method as in (c). **e,** Constant-current STM image showing a monocrystalline DCA_3Cu_2 MOF domain after annealing the sample shown in (c, d) to ~200 K (V_b = -1 V, I_t = 10 pA). Inset: Fourier transform of STM image (scalebar 0.25 nm^{-1}).

Supplementary Fig. 7 shows a large-scale STM image (size: 300x300 nm^2) of the DCA_3Cu_2 MOF on hBN/Cu(111). We do not observe any DCA-only regions across this large-scale image. There is some remaining excess Cu on

the surface (as labelled). This indicates that, although not perfect, the stoichiometry for this sample preparation was close to the required 3:2 DCA:Cu, over a large sample region. These results are similar to prior work involving growth of this MOF on graphene [12].

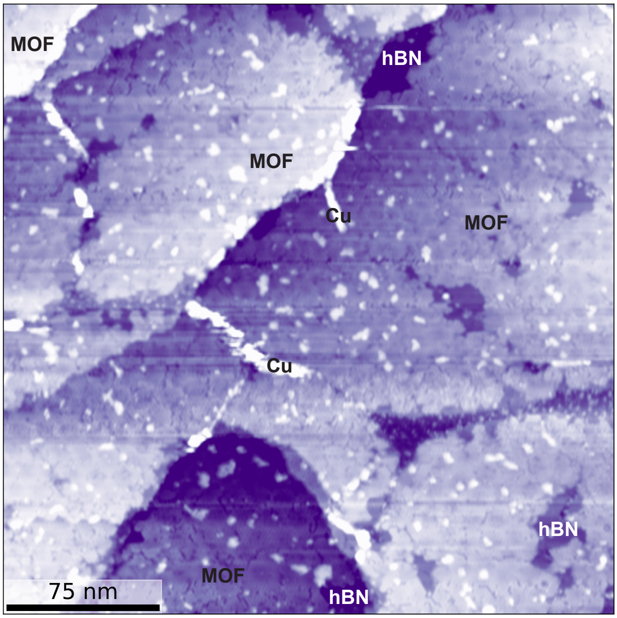

Supplementary Fig. 7: **Large-scale STM image of DCA_3Cu_2 MOF on hBN/Cu(111).** Image size: 300x300 nm^2 ($V_b = -1$ V, $I_t = 10$ pA). We observe no DCA-only regions, with only minor amounts of excess Cu. The sample preparation was close to the desired 3:2 DCA:Cu stoichiometry.

The DCA_3Cu_2 MOF was found to have a hexagonal lattice constant of 2.01 $\pm$ 0.06 nm. This suggests it is commensurate with the underlying hBN which is known to have a hexagonal lattice constant of 0.25 nm. The MOF was found to be incommensurate, however, with the hBN/Cu(111) moiré pattern. This is shown in the fast Fourier transform (FFT) in Supplementary Fig. 8b, c of the constant-current STM image in Supplementary Fig. 8a. The hBN/Cu(111) moiré pattern in this image has a lattice constant $\lambda = 10.7 \pm 0.9$ nm, at an angle of $32.0 \pm 0.5°$ with respect to the MOF lattice. The MOF appears to seamlessly grow across moiré domains of different sizes (due to different hBN domain rotations); see Supplementary Fig. 8d, and Figs. 3 and 4 of main text. This is further evidence that the MOF is incommensurate with the hBN/Cu(111) moiré pattern. Establishing whether the DCA_3Cu_2 MOF preferentially grows on hBN domains with a particular moiré periodicity is beyond the scope of this work and requires further investigation.

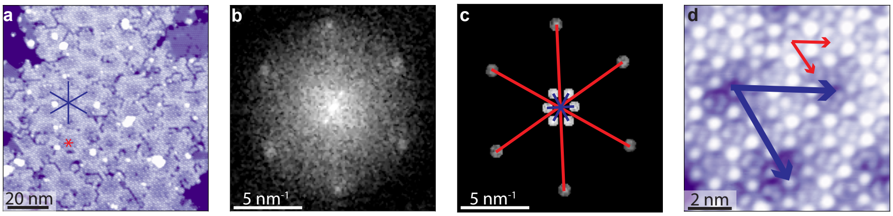

Supplementary Fig. 8: **Lattice vectors of DCA_3Cu_2 MOF on hBN/Cu(111), and of hBN/Cu(111) moiré pattern. a,** 100x100 nm^2 constant-current STM image of MOF on hBN/Cu(111) with moiré pattern clearly visible ($V_b = -1$ V, $I_t = 10$ pA). Hexagonal MOF (moiré) lattice vectors shown in red (blue, respectively). **b,** FFT of image in (a). **c,** Filtered version of FFT in (b), emphasising periodic features of MOF and hBN/Cu(111) moiré lattices. The MOF lattice vectors have a length of 2.01 $\pm$ 0.06 nm, at an angle of 32° with respect to the moiré lattice vectors which have a length of 10.7$\pm$ 0.9 nm. **d,** Constant-current STM image of MOF on hBN/Cu(111) moiré domain with a smaller period of $\sim$5 nm ($V_b = -1$ V, $I_t = 10$ pA).

Supplementary Note 7 Experimental dI/dV vs. DMFT spectral functions

Supplementary Fig. 9 shows a comparison between STS measurements performed on the DCA_3Cu_2 MOF within a pore region of the hBN/Cu(111) moiré pattern (as shown in Fig. 2 of the main text), and a DMFT spectral function (for a corresponding value of chemical potential E_F). Note that the STS measurements were performed at 4 K, whereas the DMFT calculations were performed at a temperature of 29 K (for computational convenience) and hence appear broader.

The two spectra in Supplementary Fig. 9a are asymmetric in energy: in the DCA lobe site spectrum, the UHB signature is stronger than the LHB signature; in the Cu site spectrum, the LHB signature is stronger. This may be indicative of differences in the spatial distribution of electronic states associated with these two bands. The DMFT spectral function (Supplementary Fig. 9c) does not show such asymmetry, as DMFT is unable to capture any spatially dependent orbital texture of the corresponding electronic states. The orbital texture of electronic states of Mott insulating phases is known to be poorly captured by theoretical methods in any case [13]. Note that the sum of the two dI/dV spectra in Supplementary Fig. 9a (Supplementary Fig. 9b) appears more symmetric, in agreement with the DMFT calculation.

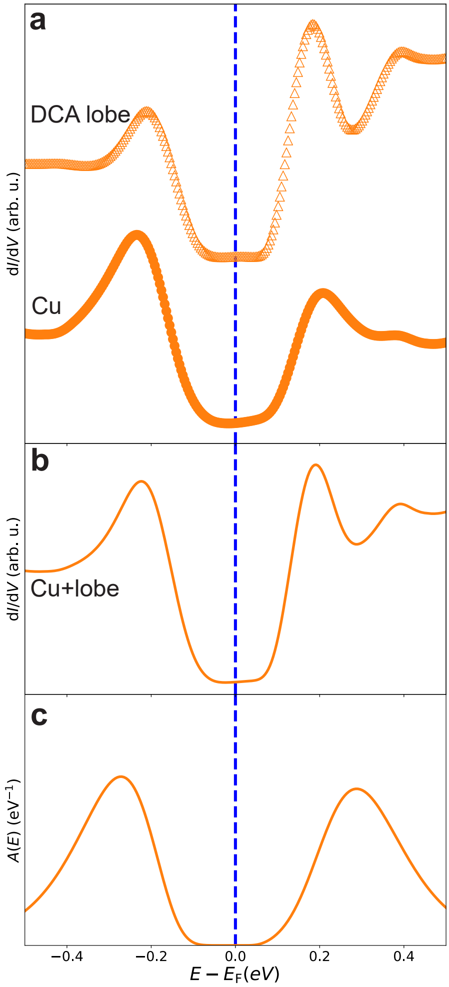

Supplementary Fig. 9: **Comparison between experimental** dI/dV **spectra and DMFT spectral function for DCA_3Cu_2 MOF at a hBN/Cu(111) moiré pore region. a,** Experimental dI/dV spectra, as shown in Fig. 2a of main text. Upper (lower) curve acquired at DCA lobe (Cu, respectively) site (tip-sample distance defined by setpoint V_b = -500 mV, I_t = 500 pA). **b,** Sum of the two spectra in (a). **c,** Spectral function, $A(E)$, predicted by DMFT, as shown in Fig. 1e of main text [$U = 0.65$ eV; E_F chosen to match electron filling predicted by DFT for MOF on hBN/Cu(111)]. Asymmetry of spectra in (a) with respect to energy may be indicative of differences in spatial distribution of electronic states associated with LHB and UHB.

Similarly, Supplementary Fig. 10 shows a comparison between STS measurements performed on the DCA_3Cu_2 MOF at a wire region of the

hBN/Cu(111) moiré pattern (as shown in Fig. 4 of the main text; for a large tip-sample distance), and a DMFT spectral function (for a corresponding value of E_F). Note the charging feature at positive bias voltage in the experimental data (due to the proximity of the LHBM to the Fermi level in the case of the wire region; see main text), which is not captured by the DMFT calculation.

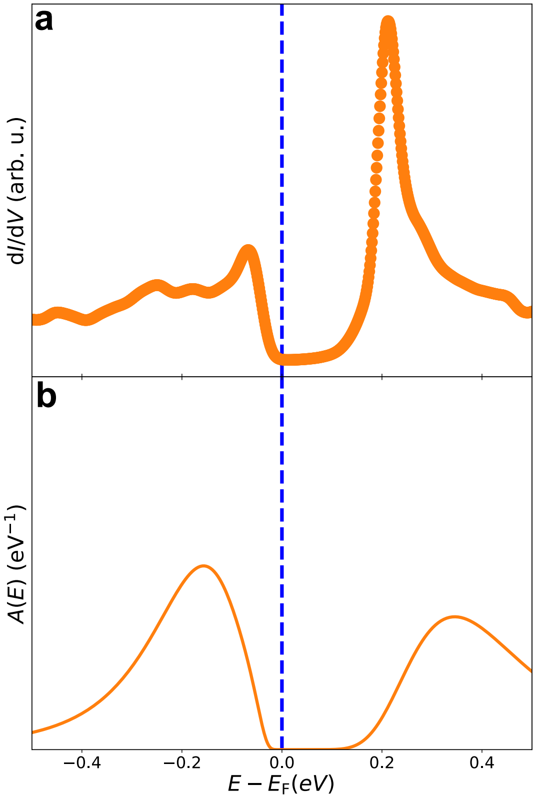

Supplementary Fig. 10: Comparison between experimental dI/dV spectrum and DMFT spectral function for DCA_3Cu_2 MOF at a moiré wire region. a, Experimental dI/dV spectrum acquired at Cu site of the MOF within wire region of hBN/Cu(111) moiré pattern (large tip-sample distance; see Fig. 4 of main text). Tip shifted 310 pm away from surface with respect to setpoint defined by $V_\mathrm{b} = 10$ mV, $I_\mathrm{t} = 10$ pA. **b,** Spectral function, $A(E)$, calculated by DMFT ($U = 0.65$ eV; E_F chosen to match LHBM position of experimental spectrum in panel a).

Supplementary Note 8 Bias-dependent STM imaging

We show a representative sample of bias-dependent constant-current STM images in the main text Fig. 2. Supplementary Fig. 11 shows a full set of these images acquired from $V_\mathrm{b} = -1$ V to 0.6 V. For $V_\mathrm{b} \lesssim$ -200 mV and $V_\mathrm{b} \gtrsim$ 200 mV, the MOF appearance remains relatively unchanged, demonstrating the characteristic spatial distribution of the electronic states contributing to the DCA_3Cu_2 kagome bands, that is, with strong intensity at the Cu sites and the ends of the DCA anthracene moieties. At bias voltages closer to the Fermi level, however, the MOF appearance changes and shows fairly uniform intensity across the DCA molecules, with reduced intensity at the Cu sites, indicative of topographic rather than electronic features. This provides strong evidence that the $\mathrm{d}I/\mathrm{d}V$ peaks at $\sim \pm 200$ mV in Fig. 2 of the main text are related to intrinsic DCA_3Cu_2 electronic states. Variation in imaging across Supplementary Fig. 11 is attributed to the influence of the hBN/Cu(111) moiré pattern, as shown in Fig. 3 of the main text.

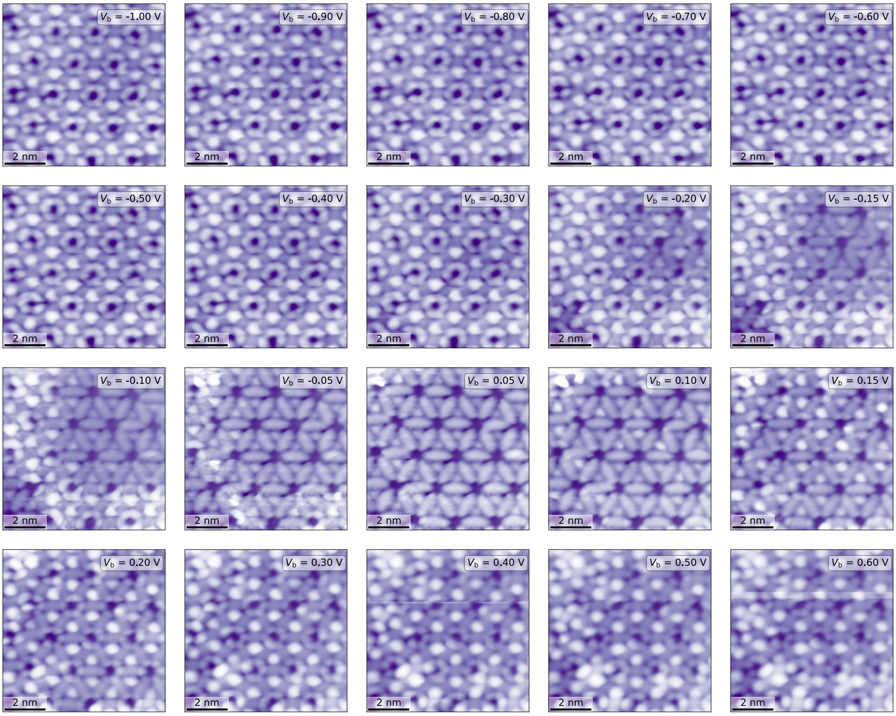

Supplementary Fig. 11: Constant-current STM images of DCA_3Cu_2 on hBN/Cu(111), at bias voltages specified in each image ($I_\mathrm{t} = 10$ pA).

Supplementary Note 9 dI/dV maps

To depict the spatial distribution of DCA_3Cu_2 MOF electronic states at different energies, we show, in the main text Fig. 2, constant-current STM images at different bias voltages V_b, rather than dI/dV maps. The spatial variation in electronic properties caused by the hBN/Cu(111) moiré pattern (main text Fig. 3) is reflected in both topographic images and dI/dV; see Supplementary Figs. 11 and 12. Constant-current STM images, which represent the integral of the local density of states (LDOS; or spectral function in general) between eV_b and the Fermi level, are however less affected than the dI/dV maps ($\propto$ LDOS) by these moiré-related variations. These constant-current STM images provide a good depiction of the intrinsic electronic character of the MOF in the electronically decoupled environment that the monolayer hBN provides.

Nonetheless, dI/dV maps are consistent with the observed spatially dependent shifts in the energy onset of bands, shown in Fig. 3 of the main text. Illustrative examples of dI/dV maps, acquired as slices of the numerical derivative of pixel-by-pixel $I(V)$ curves, are shown in Supplementary Fig. 12. These clearly show strong dI/dV intensity associated with the maxima of lower Hubbard bands (LHB) at the Cu and anthracene end sites of the DCA_3Cu_2 MOF (as shown in Fig. 2 of the main text). This intensity corresponding to the LHB appears throughout the entire hBN/Cu(111) moiré pattern at $V_b = -0.30$ V (Supplementary Fig. 12b), including at the centre of the pore region. At V_b closer to the Fermi level, however, the pore becomes progressively darker as spectra are acquired in the bandgap of the LDOS (or spectral function) in these regions.

At positive bias voltages, a similar trend is observed for the upper Hubbard band (UHB), with corresponding dI/dV intensity at the DCA lobes and Cu sites of the MOF throughout the entire hBN/Cu(111) moiré pattern at $V_b = 0.30$ V (Supplementary Fig. 12m), with the pore regions becoming progressively darker as V_b is reduced towards the Fermi level due to the energy gap (Supplementary Fig. 12h-l). The trend is less obvious for positive biases than for negative, however, as the dI/dV signal at wire regions show charging features in this energy range (as shown in Fig. 4 of main text).

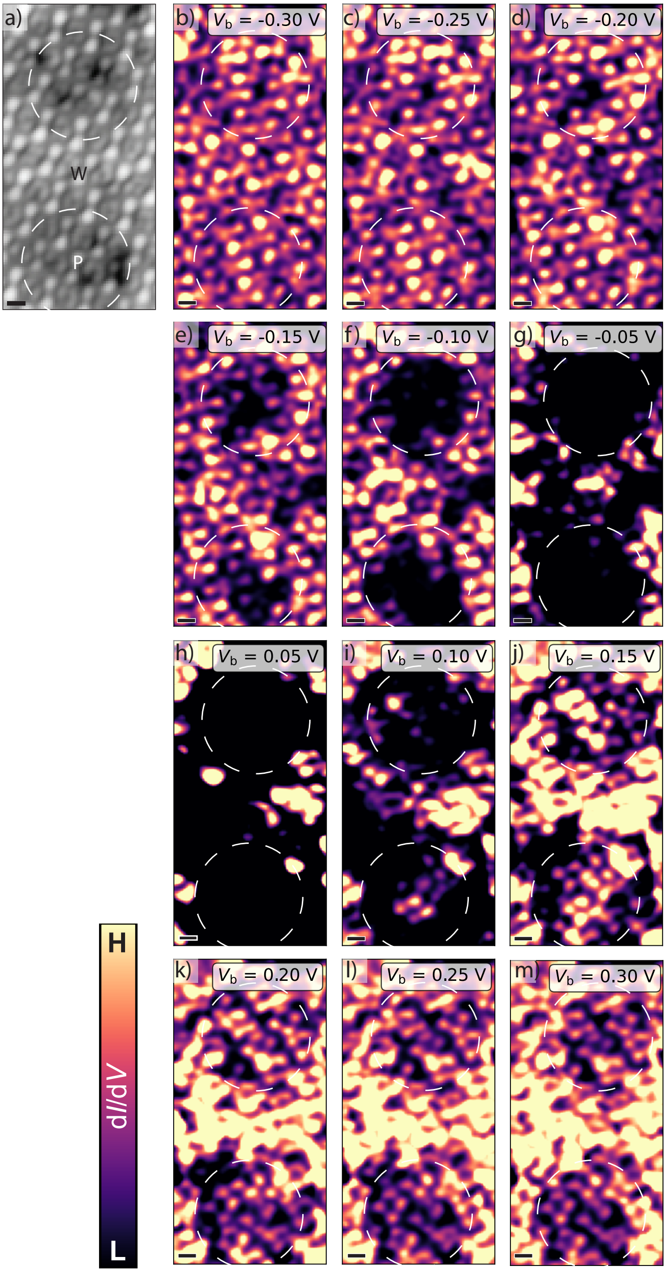

Supplementary Fig. 12: dI/dV maps of DCA_3Cu_2/hBN/Cu(111). a, Constant-current STM image of DCA_3Cu_2/hBN/Cu(111) containing two pore (P) regions (dashed white circles) separated by a wire (W) region ($V_b = -1$ V, $I_t = 10$ pA). **b-m,** dI/dV maps at the bias voltages specified in each panel, acquired in the same region as (a). At each pixel, the tip height was stabilised 225 pm further away from the surface with respect to a setpoint of $V_b = 10$ mV, $I_t = 10$ pA, before $I(V)$ acquisition. Scale bars: 1 nm.

Supplementary Note 10 STS measurements at MOF high-symmetry points

In the main text, we focus on DCA lobe and Cu sites of the MOF to highlight its electronic properties, since the electronic states associated with the near-Fermi kagome bands have predominantly DCA LUMO character (with some Cu character), and such states are mostly localised at these sites [12, 14]. STS measurements at other high-symmetry locations within the MOF (e.g., DCA molecule centre; MOF pore site – to not confuse with hBN/Cu(111) moiré pore region) are displayed in Supplementary Fig. 13b. These MOF pore (yellow) and DCA centre (blue) spectra appear qualitatively consistent with the DCA lobe (green) and Cu (purple) site spectra, with a gap at the Fermi level between LHB and UHB. Note that the dI/dV signal associated with LHB and UHB is weaker at MOF pore and DCA centre sites than at DCA lobe and Cu sites. The dI/dV signal at MOF pore and DCA centre sites is given by signatures of the MOF near-Fermi electronic structure which are strongest at DCA lobe and Cu sites.

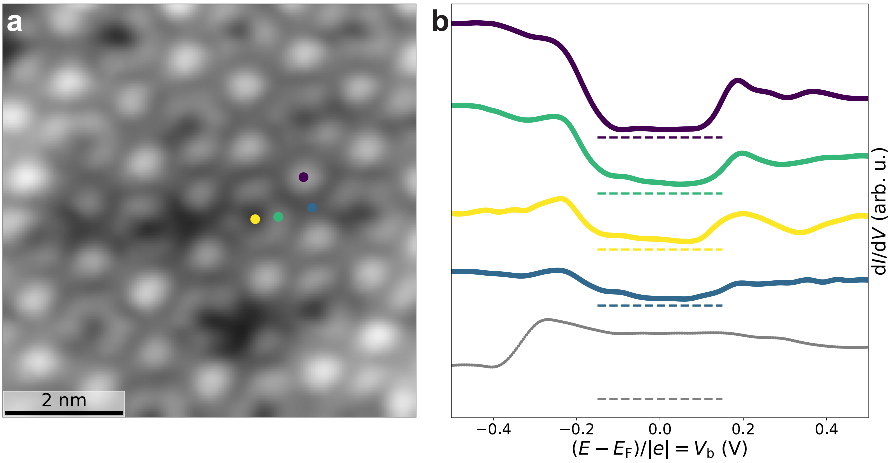

Supplementary Fig. 13: **STS measurements at MOF high-symmetry locations within a pore region of the hBN/Cu(111) moiré pattern.** **a,** STM image of DCA_3Cu_2 MOF on hBN/Cu(111) ($V_b = -1$ V, $I_t = 10$ pA). **b,** STS measurements performed at positions corresponding to coloured markers in (a). Grey curve: reference spectrum acquired upon bare hBN/Cu(111). Curves offset for clarity. Dashed lines indicate position of dI/dV = 0. Tip height stabilised 150 pm further away from the surface with respect to setpoint of $V_b = 10$ mV, $I_t = 10$ pA.

Supplementary Note 11 Electronic influence of defects

The DCA_3Cu_2 MOF domains on hBN/Cu(111) show good monocrystallinity, arguably given by the good match between MOF and hBN lattices. Nonetheless, a number of defects such as domain edges and boundaries, vacancies, and cracks can be observed (see main text Fig. 1a). To investigate the influence of these defects on the electronic properties of the MOF, we conducted STS measurements at the locations of these defects.

STS measurements performed at the edge of a DCA_3Cu_2 MOF domain (within a pore region of the hBN/Cu(111) moiré) are shown in Supplementary Fig. 14. At the very edge of the domain, DCA molecules are coordinated to only one Cu atom (rather than two as is the case within the bulk). Spectra acquired at these positions exhibit a LHB feature, similar to spectra within the MOF domain reported elsewhere in this manuscript. However, these spectra feature heavily reduced UHB weight and an additional peak at ~0.5-0.7 V, not seen for spectra within the MOF domain. This additional peak is reminiscent of the DCA LUMO for purely organic films of DCA molecules on hBN/Cu(111) [11]. The peak observed here appears at a lower energy, however. We therefore claim that the DCA molecules at the edges of the MOF domain, coordinated to just one Cu atom, exhibit electronic properties which are a mixture of typical DCA_3Cu_2 properties and those of DCA-only domains. Importantly, just one unit cell away from the domain edges, the DCA_3Cu_2 properties are identical to those within a MOF domain region without defects, with clear LHB and UHB signatures, indicating that these domain edges do not significantly affect the electronic characteristics of the MOF domain bulk discussed throughout the manuscript.

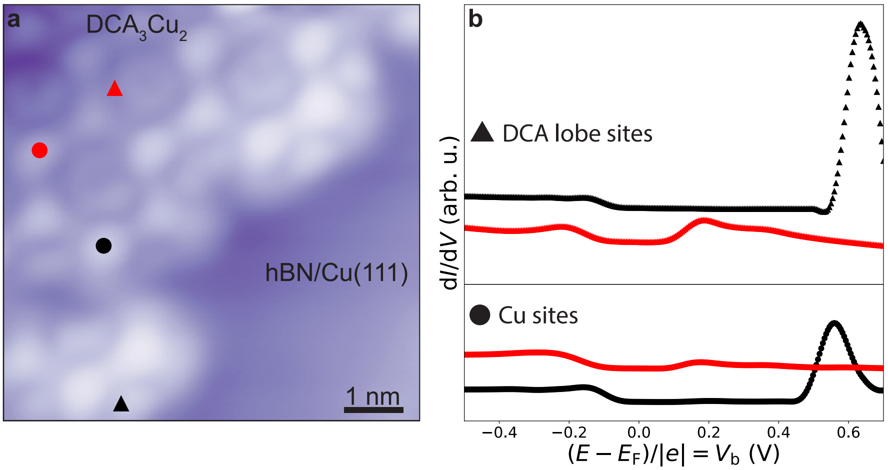

Supplementary Fig. 14: **STS measurements at the edge of a DCA_3Cu_2 MOF domain. a,** STM image of the edge of a MOF domain ($V_b = -1$ V, $I_t = 10$ pA). **b,** STS measurements at Cu sites (circle markers) and DCA lobe sites (triangle markers) both at the edge of the MOF domain (black) and within the MOF domain (red). Curves offset for clarity. Setpoints: $V_b = -500$ mV, $I_t = 100$ pA.

We observed similar electronic properties at boundaries between DCA_3Cu_2 MOF domains. These domain boundaries can be observed in a number of STM images presented in this work and appear as dark "cracks" within the MOF domains (Supplementary Fig. 15a). Similar to the edges of MOF domains, these domain boundaries feature DCA molecules coordinated to just one Cu atom. STS measurements performed at one of these domain boundaries are shown in Supplementary Fig. 15b. As was the case for the edge of the MOF domain, the spectrum acquired at the domain boundary exhibits reduced UHB weight and an additional peak at ∼0.55 V. This again suggests that the electronic properties of the singly Cu-coordinated DCA molecules here are a mixture of Mott insulating DCA_3Cu_2 and the LUMO of the DCA-only phase.[11] Once again, just one unit cell away from these boundaries, the electronic properties are identical to those within a MOF domain region without defects, indicating that domain boundary defects do not significantly affect the intrinsic electronic properties of a MOF domain bulk.

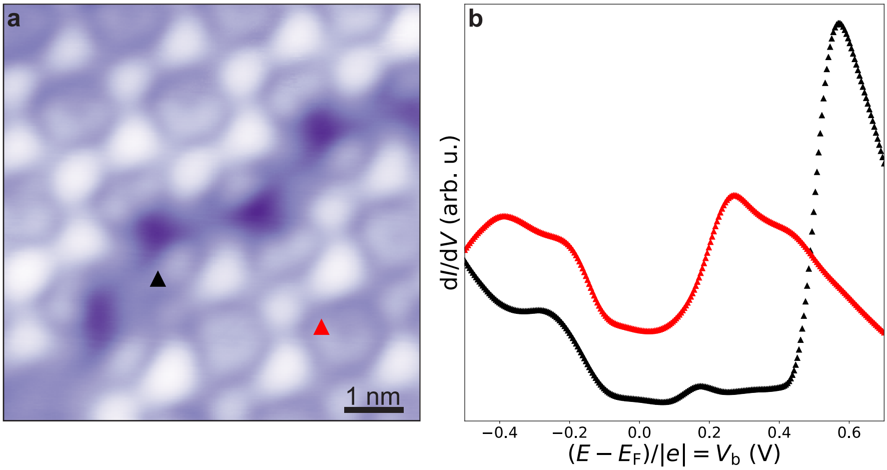

Supplementary Fig. 15: **STS measurements at DCA_3Cu_2 MOF domain boundary. a,** STM image showing a MOF domain boundary (V_b = −1 V, I_t = 10 pA). **b,** STS measurements at DCA lobe sites, both at the MOF domain boundary (black) and within the MOF domain away from the boundary (red). Curves offset for clarity. Setpoints: V_b = −500 mV, I_t = 100 pA.

Finally, we also considered the influence of Cu vacancy defects which were present in some MOF domains. Two spectra at Cu sites of the DCA_3Cu_2 MOF in proximity to a Cu vacancy (green and orange), and one spectrum at the Cu vacancy site (blue) are shown in Supplementary Fig. 16. The three spectra are qualitatively similar, featuring a pronounced energy gap across the Fermi level indicating the Mott insulating phase. The blue spectrum, however, has noticeably weaker signal at the LHB and UHB. Overall, the vacancy defect does not appear to significantly perturb the MOF's electronic properties.

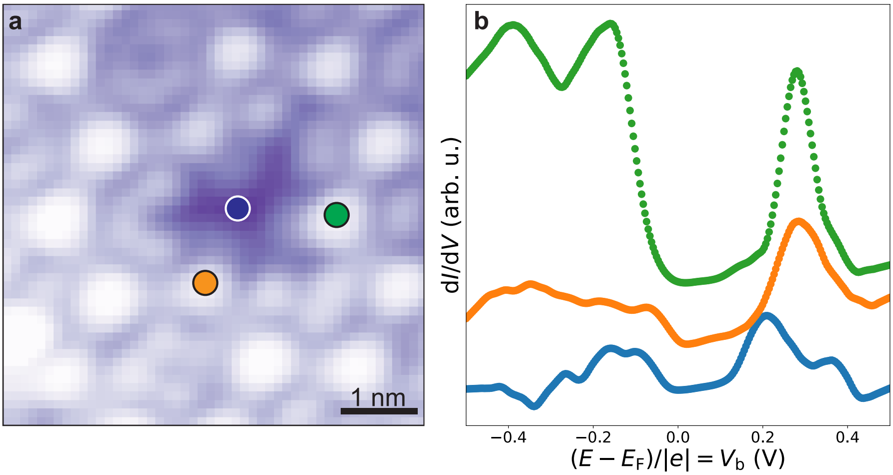

Supplementary Fig. 16: **STS measurements at Cu vacancy defect within a DCA_3Cu_2 MOF domain. a,** STM image showing a Cu vacancy defect ($V_b = -1$ V, $I_t = 10$ pA). **b,** STS measurements at Cu sites in proximity of Cu vacancy (green and orange), and at Cu vacancy (blue). Curves offset for clarity. Setpoints: 135pm further away from the surface with respect to a setpoint of $V_b = 10$ mV, $I_t = 10$ pA.

Supplementary Note 12 Broad energy-range STS measurements

In the main text, we focus on STS measurements in an energy window from −0.5 to 0.5 eV. This energy window was selected based on DFT predictions according to which the near-Fermi kagome MOF bands are well isolated and separated in energy (>1 eV) from other bands. To verify this assumption, we conducted STS measurements over a broader energy window, from -1 V to 1 V, at a DCA lobe site of the DCA_3Cu_2 MOF within a pore region of the hBN/Cu(111) moiré pattern (Supplementary Fig. 17). The LHB and UHB features, as well as the ∼200 meV energy gap, are evident in the MOF spectrum. No other significant features can be observed in this energy window. This is consistent with the theoretical predictions and justifies our focus on the near-Fermi energy window within the main text.

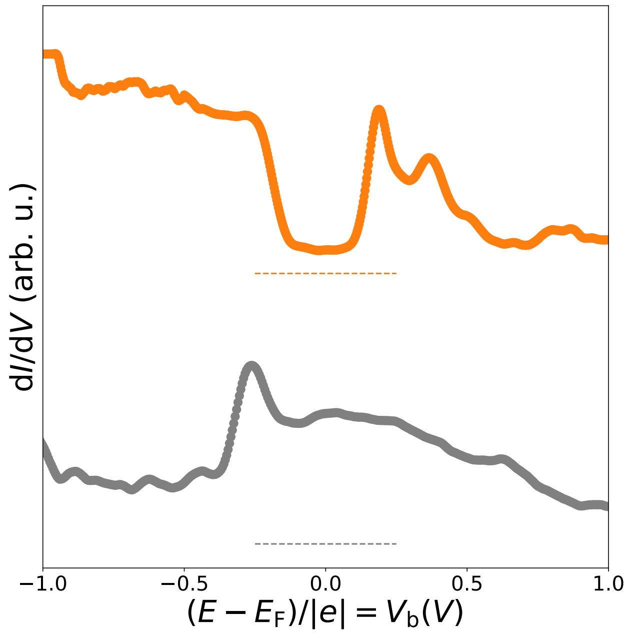

Supplementary Fig. 17: **STS measurements over broad energy window.** Orange curve: DCA lobe site of MOF within pore region of hBN/Cu(111) moiré pattern. Grey curve: bare hBN/Cu(111) reference spectrum. Spectra normalised and offset for clarity. Orange curve acquired in two parts: setpoint of $V_\mathrm{b} = -1$ V, $I_\mathrm{t} = 100$ pA from -1 V to -500 mV; setpoint of $V_\mathrm{b} = -500$ mV, $I_\mathrm{t} = 100$ pA for the remaining bias range. Grey curve setpoint: $V_\mathrm{b} = -2$ V, $I_\mathrm{t} = 100$ pA.

Supplementary Note 13 Determination of band edges

In Figs. 3 and 4 of the main text, MOF band edges are tracked as a function of position on the hBN/Cu(111) moiré pattern and of tip-sample distance, respectively. Determining precisely the bias voltage V_b corresponding to these band edges can be challenging due to a non-zero $\mathrm{d}I/\mathrm{d}V$, even in the gap region, resulting from the underlying Cu(111) LDOS.

We therefore determined such band edges by finding a V_b value, V_b^*, corresponding to a local minimum of $\mathrm{d}I/\mathrm{d}V$ in a small window about $V_\mathrm{b} = 0$ (i.e., $\mathrm{d}^2I/\mathrm{d}V^2 = 0$). We then determined the lower (upper) Hubbard band edge as the value of V_b closest to and below (above) V_b^*, for which $|\mathrm{d}^2I/\mathrm{d}V^2(V_\mathrm{b})| > \epsilon$, where ϵ represents a specified threshold. A consequence of this method was that lower Hubbard band edges were sometimes located at $V_\mathrm{b} > 0$ V; we claim this is consistent with the observed reduction of the hole creation energy barrier at the wire regions of the hBN/Cu(111) moiré pattern (suggesting that the

Mott phase collapses in these regions, consistent with the spectral functions predicted by DMFT for different values of E_F in Supplementary Fig. 2). An example of the application of this method is shown in Supplementary Fig. 18.

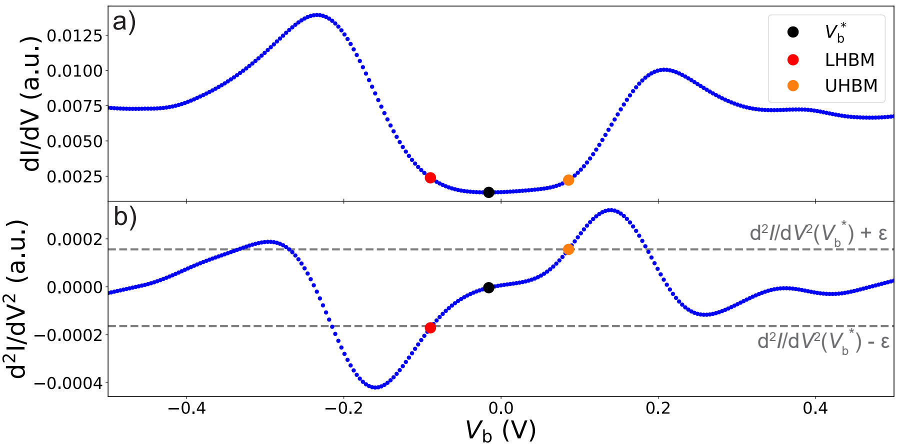

Supplementary Fig. 18: **Method for determining band edges in dI/dV spectra. a,** dI/dV spectrum obtained via the numerical derivative of an $I(V)$ curve at a Cu site of DCA_3Cu_2 MOF on hBN/Cu(111) (setpoint: $V_b = -500$ mV, $I_t = 500$ pA). **b,** d^2I/dV^2 spectrum for the same data as in (a). Band edges are determined by the values of V_b near V_b^*, a local minimum of dI/dV near the Fermi level, for which $|d^2I/dV^2(V_b)| > \epsilon$ (where ϵ is a specified threshold).

The determination of band edges for the DMFT spectral functions $A(E)$ was simpler, since $A(E) \to 0$ for energies E within the bandgap. This energy gap was identified as a continuous energy region around the chemical potential E_F associated with values of the (normalised) spectral function smaller than some threshold value. The threshold was chosen as 5×10^{-3} t^{-1} (0.1 eV^{-1}). This value set the band edges at energy positions agreeing with those determined by the above method for experimental spectra.

Supplementary Note 14 Moiré-dependent STS measurements at DCA lobe sites of DCA_3Cu_2 MOF

In Supplementary Fig. 3 of the main text, we showed STS measurements at Cu sites of the DCA_3Cu_2 MOF, at different locations of the hBN/Cu(111) moiré pattern, from pore to wire to pore regions. Equivalent measurements performed at the same hBN/Cu(111) moiré pattern as in Fig. 3, with similar tunneling parameters (e.g., intermediate tip-sample distances), but at DCA lobe sites

are shown in Fig. 19. This figure shows the same trend as Fig. 3. The LHB and UHB shift in energy across the electronically corrugated hBN/Cu(111) moiré pattern. At the wire regions, the energy gap of ~200 meV at the Fermi level is no longer observed, and the Fermi-level dI/dV signal increases. These findings are consistent with our interpretation that the DCA_3Cu_2 MOF is a Mott insulator on the hBN/Cu(111) substrate, and that a combination of template- and tip-induced gating can depopulate the kagome bands to induce a local Mott metal-insulator transition.

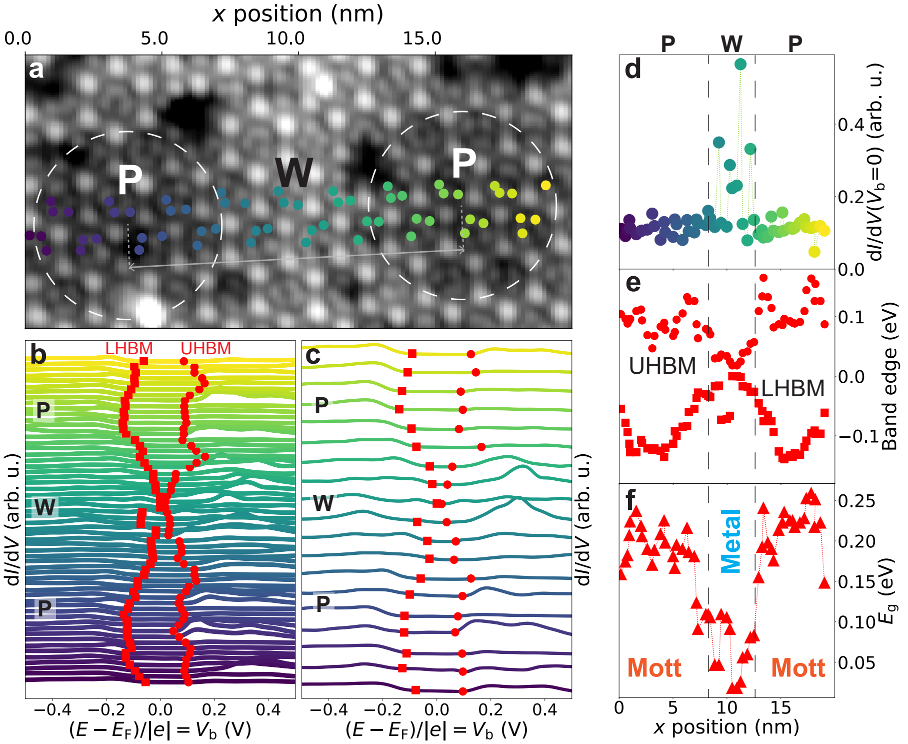

Supplementary Fig. 19: **STS measurements at DCA lobe sites of DCA_3Cu_2 MOF at different locations of the hBN/Cu(111) moiré pattern. a,** STM image of MOF (V_b = -1 V, I_t = 10 pA). White dashed circles (P): hBN/Cu(111) moiré pore regions, separated by wire region (W). Grey arrow indicates moiré period $\lambda \approx 12.5$ nm. **b,** dI/dV spectra acquired at MOF DCA lobe sites, at positions indicated by coloured markers in (a). Tip 190 pm further from surface with respect to setpoint V_b = 10 mV, I_t = 10 pA). Energy gap $E_g \approx 200$ meV at Fermi level at P regions, with no Fermi-level gap at W region (LHBM: lower Hubbard band maximum; UHBM: upper Hubbard band minimum). **c,** Selection of spectra in (b), plotted for clarity. **d,** dI/dV signal at Fermi level ($V_b = 0$) as a function of position x with respect to hBN/Cu(111) moiré pattern, for each spectrum in (b), showing increased signal within the W region. **e, f,** LHBM (squares), UHBM (circles) and energy gap E_g (triangles), as a function of x position, extracted from (b).

Supplementary Note 15 STS measurements for hBN/Cu(111) moiré domains with different periods

Due to different rotational domains of hBN on Cu(111), moiré patterns with different periods are possible. As described in Supplementary Note 6, the DCA_3Cu_2 MOF grows seamlessly across moiré patterns with different periods.

To assess the influence of different hBN/Cu(111) moiré periods on the electronic properties of the DCA_3Cu_2 MOF, we performed STS measurements equivalent to those shown in Fig. 3 of the main text (for a hBN/Cu(111) moiré domain with a period $\lambda = 12.5$ nm) on two additional moiré patterns with periods of $\lambda \approx 10$ nm and $\lambda \approx 5$ nm (Supplementary Figs. 20, 21).

As in Fig. 3 of the main text, both Supplementary Figs. 20 and 21 show the LHB and UHB shifting in energy as a function of position on the electronically corrugated moiré pattern. The LHBM varies sinusoidally from pore to wire to pore region, with the period of the moiré pattern (Supplementary Figs. 20d, 21d). These spectra are gapped at the Fermi level at the pore regions (therefore insulating), with no gap at the Fermi level (Supplementary Figs. 20e, 21e) and a pronounced increase in Fermi-level $\mathrm{d}I/\mathrm{d}V$ signal at the wire regions (therefore metallic; Supplementary Figs. 20c, 21c).

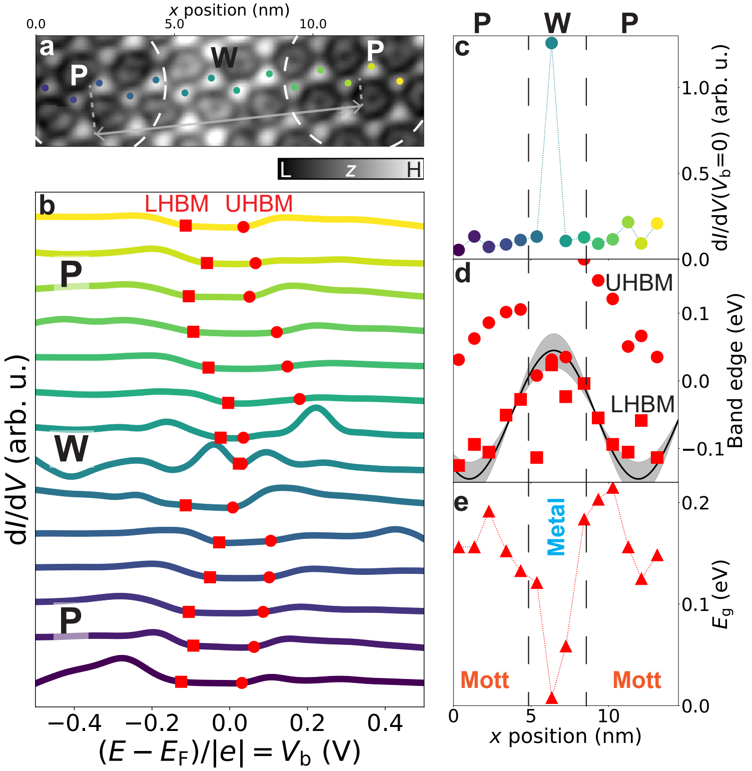

Supplementary Fig. 20: **STS measurements at Cu sites of MOF on hBN/Cu(111) domain with moiré period $\lambda \approx 10$ nm. a,** STM image of MOF (V_b = -1 V, I_t = 10 pA). White dashed circles (P): hBN/Cu(111) moiré pore regions, separated by wire region (W). Grey arrow indicates moiré period. **b,** dI/dV spectra acquired at MOF Cu sites, at positions indicated by coloured markers in (a). Tip 190 pm further from surface with respect to setpoint V_b = 10 mV, I_t = 10 pA. Energy gap $E_g \approx 200$ meV at Fermi level for P regions, vanishing at W region (LHBM: lower Hubbard band maximum; UHBM: upper Hubbard band minimum). **c,** dI/dV signal at Fermi level ($V_b = 0$) as a function of position x on hBN/Cu(111) moiré pattern, extracted from spectra in (b), showing increased signal within W region. **d, e,** LHBM (squares), UHBM (circles) and energy gap E_g (triangles) as a function of x position, extracted from (b). Black line in (d): expected LHBM variation due to local moiré work function modulation for this moiré period [3]. Grey shaded area: uncertainty.

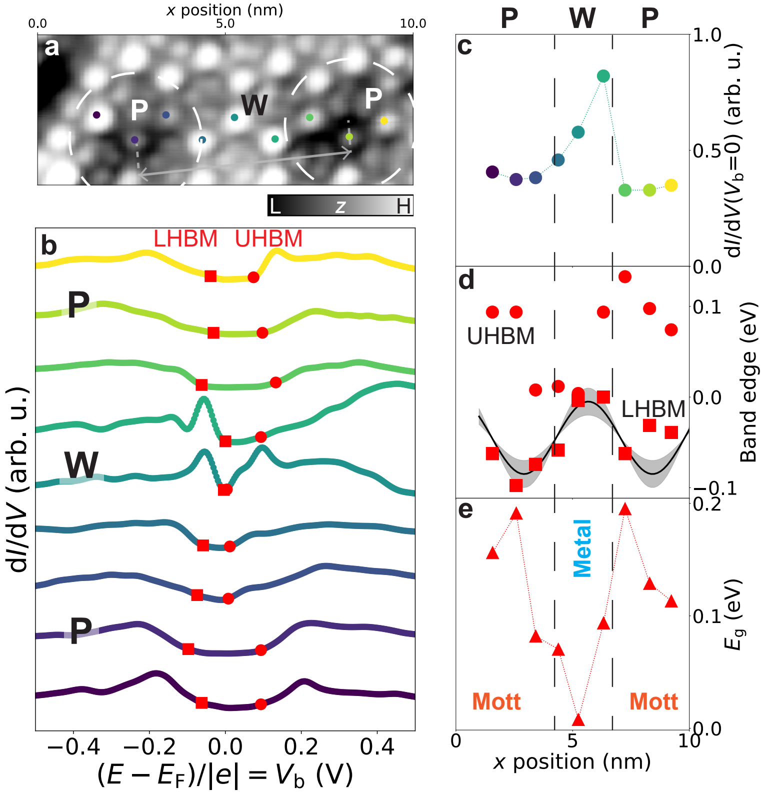

Supplementary Fig. 21: **STS measurements at Cu sites of MOF on hBN/Cu(111) domain with moiré period $\lambda \approx$ 5 nm. a,** STM image of MOF (V_b = -1 V, I_t = 10 pA). White dashed circles (P): hBN/Cu(111) moiré pore regions, separated by wire region (W). Grey arrow indicates moiré period. **b,** dI/dV spectra acquired at MOF Cu sites, at positions indicated by coloured markers in (a). Tip 190 pm further away from surface with respect to setpoint V_b = 10 mV, I_t = 10 pA. Energy gap $E_\mathrm{g} \approx$ 200 meV at Fermi level for P regions, vanishing at W region (LHBM: lower Hubbard band maximum; UHBM: upper Hubbard band minimum). **c,** dI/dV signal at the Fermi level (V_b = 0) as a function of position x on hBN/Cu(111) moiré pattern, extracted from spectra in (b), showing increased signal within W region. **d, e,** LHBM (squares), UHBM (circles) and energy gap E_g (triangles) as a function of x position, extracted from (b). Black line in (d): expected LHBM variation due to local moiré work function modulation for this moiré period [3]. Grey shaded area: uncertainty.

In Fig. 3 of the main text, the dI/dV spectra with a gap at the Fermi level show a sinusoidal variation of the Hubbard band edges that follows the

variation of the local work function given by the hBN/Cu(111) moiré pattern. For a hBN/Cu(111) moiré pattern with a periodicity of ∼12.5 nm (as in the main text), such local work function variation from pore to wire region is of ∼200 meV [3]. For the two moiré patterns in Supplementary Figs. 20 and 21, the energy variation of the LHBM is smaller: ∼190 meV for $\lambda \approx 10$ nm and ∼100 meV for $\lambda \approx 5$ nm. These values are in good agreement with the local work function modulation for these moiré periods (black curves with grey uncertainty regions in Supplementary Figs. 20d and 21d) [3]. These findings are summarised in Supplementary Fig. 22. The black markers show the expected local work function modulation for different hBN/Cu(111) moiré periods [3]. The red markers show the experimentally observed variation in LHBM energy between pore and wire regions, for $\lambda \approx 10$ nm (Supplementary Fig. 20), $\lambda \approx 5$ nm (Supplementary Fig. 21) and $\lambda \approx 12.5$ nm (main text Fig. 3). Our experimental LHBM energy shifts lie within the uncertainty of the moiré local work function variation. This agreement validates our interpretation of the Hubbard band shifts resulting from the electronically corrugated hBN/Cu(111) moiré pattern. Notably, the electronic properties of the MOF are consistent for hBN/Cu(111) moiré domains with significantly different periods.

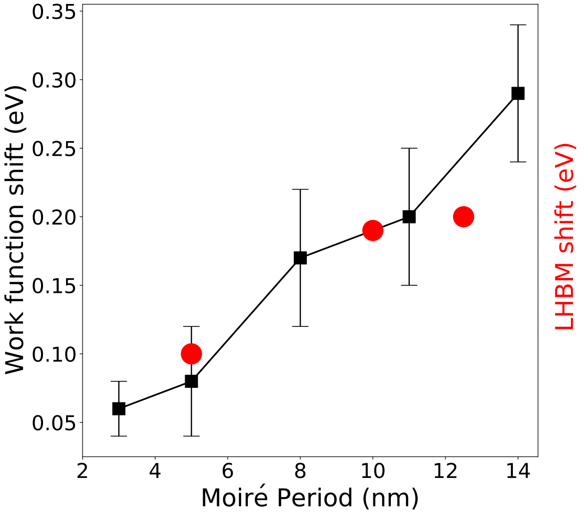

Supplementary Fig. 22: **Comparison between local work function modulation and LHBM variation for different moiré periods.** Black markers: local work function shift (and uncertainties) for different moiré periods, from prior literature [3]. Red markers: Experimentally observed variations in LHBM energy for the three different moiré periods considered in this work.

Supplementary Note 16 Charging signatures

In the dI/dV spectra in Fig. 4 of the main text, we observe narrow peaks whose energy position vary linearly as a function of tip-sample distance $\Delta z + z_0$. Based on a double-barrier tunnel junction (DBTJ) model, we attribute these features to charging of intrinsic electronic states of the MOF. Further evidence that these features are related to charging phenomena is provided by the presence of charging rings in dI/dV maps. An example is shown in Supplementary Fig. 23. At a bias voltage $V_\mathrm{b} \approx 0.05$ V (Supplementary Fig. 23b), we observe dI/dV intensity at the centre of a MOF DCA molecule, within a wire region of the hBN/Cu(111) moiré pattern. As V_b increases (Supplementary Fig. 23c-m), this dI/dV intensity moves gradually towards the edges of the DCA molecule, with the shape of a ring, consistent with the expected charging ring behaviour [12, 15].

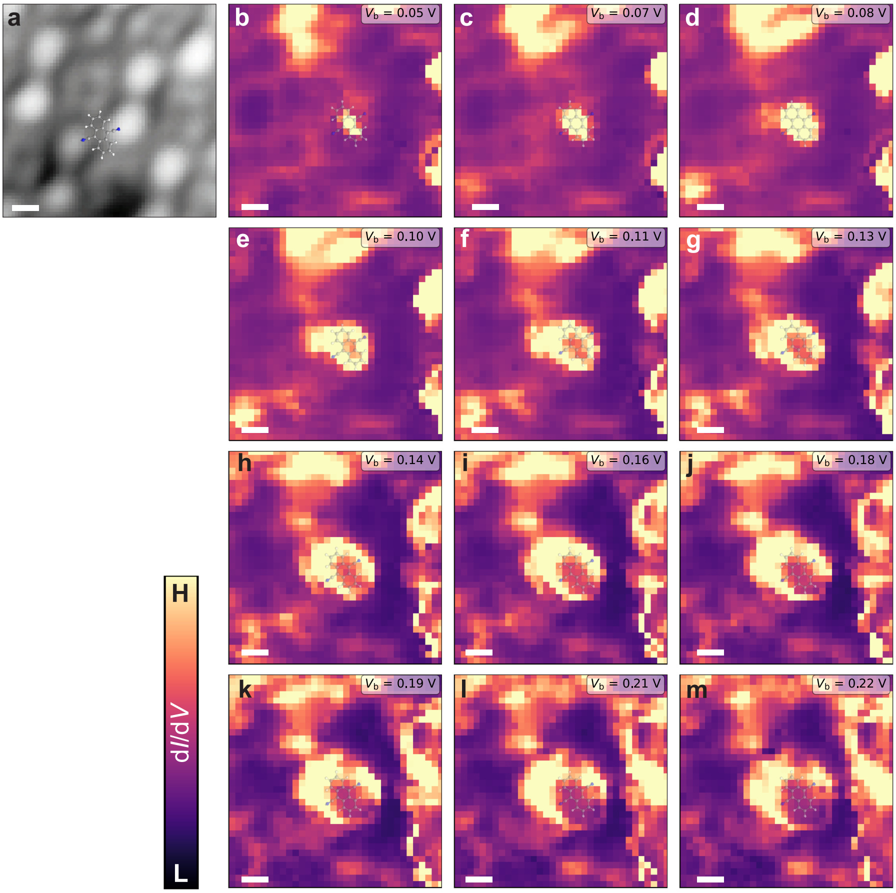

Supplementary Fig. 23: dI/dV maps of DCA$_3$Cu$_2$/hBN/Cu(111): charging ring. a, STM image of DCA$_3$Cu$_2$ at a wire region of hBN/Cu(111) moiré pattern ($V_b = -1$ V, $I_t = 10$ pA). **b-m,** dI/dV maps of region in (a), at indicated bias voltages V_b, obtained via numerical derivative of pixel-by-pixel $I(V)$ curves. At each pixel, the tip-sample distance was stabilised 300 pm further away from the surface relative to a setpoint of $V_b = 10$ mV, $I_t = 10$ pA, before $I(V)$ acquisition. Scale bars: 1 nm.

Supplementary Note 17 Charging features in MOF dI/dV at pore-wire boundary of moiré pattern

In Fig. 3b of the main text, some MOF dI/dV spectra acquired at locations in between the hBN/Cu(111) moiré pore and wire regions featured peaks at biases of ∼0.4 eV. These peaks are not captured by the corresponding DMFT calculations in Fig. 3d. We conducted further dI/dV measurements as a function of

tip-sample distance $\Delta z + z_0$ at a MOF Cu site at such boundary locations of the moiré pattern (Supplementary Fig. 24). The dI/dV spectra in Supplementary Fig. 24b exhibit a narrow peak at $V_\mathrm{b} > 0.4$ V for intermediate Δz values, progressively shifting to lower energies with decreasing Δz. This is very similar to the tip-induced charging behaviour due to the DBTJ effect demonstrated in Fig. 4. This provides an explanation for the DMFT spectral functions in Fig. 3d not showing such a peak, since DMFT does not capture tip-induced phenomena. Note that, for the range of tip-sample distances explored, we did not observe a transition to a metal-like phase at this specific acquisition site.

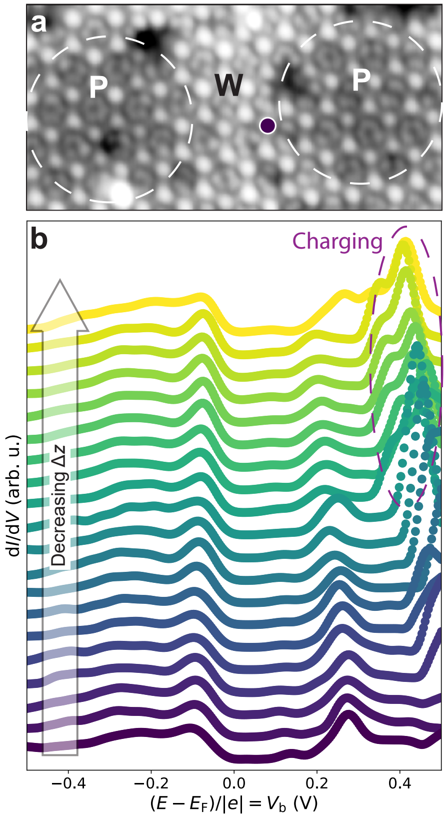

Supplementary Fig. 24: **Charging features at a pore-wire boundary of the moiré pattern. a,** STM image of MOF/hBN/Cu(111) showing two pore regions and one wire region of the hBN/Cu(111) moiré pattern ($V_\mathrm{b} = -1$ V, $I_\mathrm{t} = 10$ pA). **b,** dI/dV spectra for different tip-sample distance changes Δz, acquired at MOF Cu site, as indicated in (a). Spectra normalised and offset for clarity. Setpoints range from 250 pm further from surface (bottom curve) to 105 pm further from surface with respect to a setpoint of $V_\mathrm{b} = 10$ mV, $I_\mathrm{t} = 10$ pA.

Supplementary Note 18 Estimate of tip work function

We estimated the tip work function, Φ_t, for the tip used to acquire the $dI/dV(\Delta z)$ spectra in Fig. 4 of the main text, by conducting $I_t(\Delta z)$ spectroscopy on the MOF at a pore region of the hBN/Cu(111) moiré pattern. Assuming a square tunnelling barrier, the tunnelling current I_t as a function of changing tip-sample distance, Δz, is given by:

$$I_t(\Delta z) = I_0 \exp(-\kappa \Delta z), \tag{4}$$

where I_0 represents the current at an initial tip-sample distance z_0, and

$$\kappa = 2\sqrt{\frac{2m_e\Phi_{av}}{\hbar^2}}, \tag{5}$$

where m_e is the electron mass, $\hbar$ the reduced Planck's constant and Φ_{av} represents the average tunneling barrier given by:

$$\Phi_{av} = \frac{\Phi_t + \Phi_s - eV_b}{2}, \tag{6}$$

where Φ_t and Φ_s are the tip and sample work functions, respectively, V_b is the applied bias voltage, and e the charge of an electron.

The moiré pattern of hBN/Cu(111) is known to have a maximum work function modulation of ~0.3 eV, with a work function of ~4.10 eV at the wire region and ~3.80 eV at the pore region centre [16–18]. This work function modulation depends on the specific moiré periodicity λ, which for Figs. 3 and 4 of the main text is $\lambda \approx 12.5$ nm, resulting in a work function modulation of ~0.2 eV [3]. Based on this, we estimate that, for the specific domain of interest, the MOF/hBN/Cu(111) system has an average work function of 3.95 eV (a value also consistent with DFT findings [6]), with $\Phi_s = 3.85$ eV at the pore region centre and $\Phi_s = 4.05$ eV at the wire region. Using this value for the pore region centre, we extracted κ from the slope of $\ln[I_t(\Delta z)/I_0] = -\kappa\Delta z$ in Supplementary Fig. 25, which allowed us to estimate a tip work function of $\Phi_t = 4.84$ eV.

Alternatively, from the fits in Fig. 4e of the main text, we extracted a difference in work function between tip and sample at the wire region of 0.36 eV. Taking $\Phi_s = 4.05$ eV at the wire region, this implies a value of $\Phi_t = 4.41$ eV. The two estimates of Φ_t differ slightly, and we emphasise that they should be treated as approximate values. Importantly, however, both imply that $\Phi_t > \Phi_s$ at both the pore and wire regions of the moiré pattern, consistent with the Δz-dependence of the observed tip-induced gating in Fig. 4 of the main text and Supplementary Fig. 27.

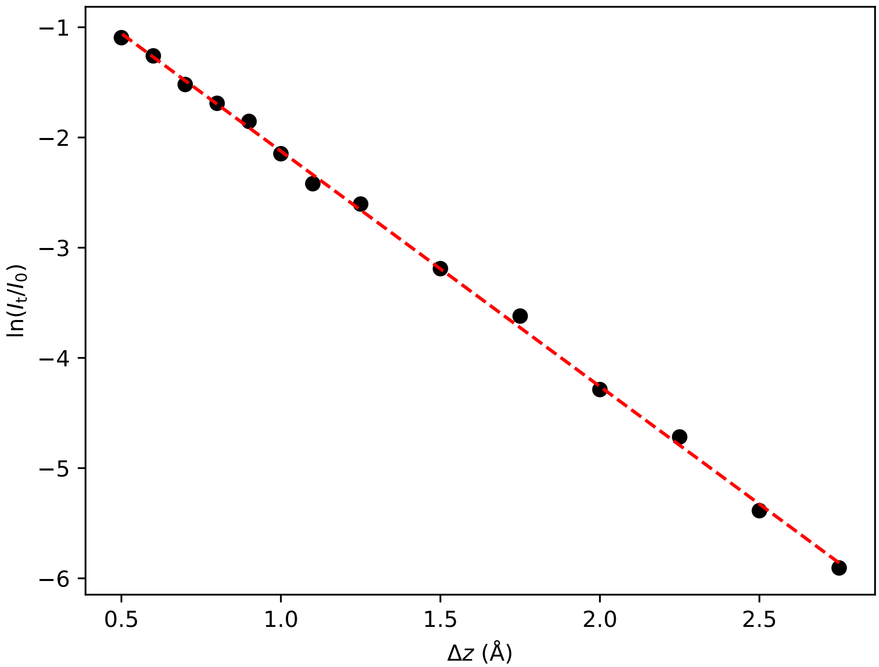

Supplementary Fig. 25: $\ln(I_\mathrm{t}/I_0)$ as a function of tip-sample distance change Δz, performed at a DCA anthracene end site of DCA_3Cu_2 MOF on hBN/Cu(111) at a bias of $V_\mathrm{b} = 10$ mV (pore region; hBN/Cu(111) moiré pattern periodicity $\lambda = 12.5$ nm; see red marker in Supplementary Fig. 27c for location). Dashed red line: fit using Supplementary Eq. (4), allowing for the extraction of κ. STM setpoint: $V_\mathrm{b} = 10$ mV, $I_0 = 10$ pA, determining initial tip-sample distance $\Delta z = 0$ Å.

Supplementary Note 19 DBTJ model: energy diagrams

In Fig. 4 of the main text and Supplementary Fig. 26, we show schematics and energy level diagrams of the double-barrier tunnel junction (DBTJ), consisting of tip, vacuum tunnelling barrier between tip and MOF, MOF, hBN tunnelling barrier between MOF and Cu(111) substrate, and Cu(111) substrate, at different tip-sample distances $\Delta z + z_0$ and bias voltages V_{b}. With $\Phi_{\mathrm{t}} > \Phi_{\mathrm{s}}$ (see above), the effective electron filling of the kagome bands of the MOF decreases as Δz decreases. This process sequentially drives the Mott insulator to metal to trivial insulator transition. The MOF can become charged due to resonant electron transfer when MOF electronic states align with the Cu(111) substrate Fermi level.

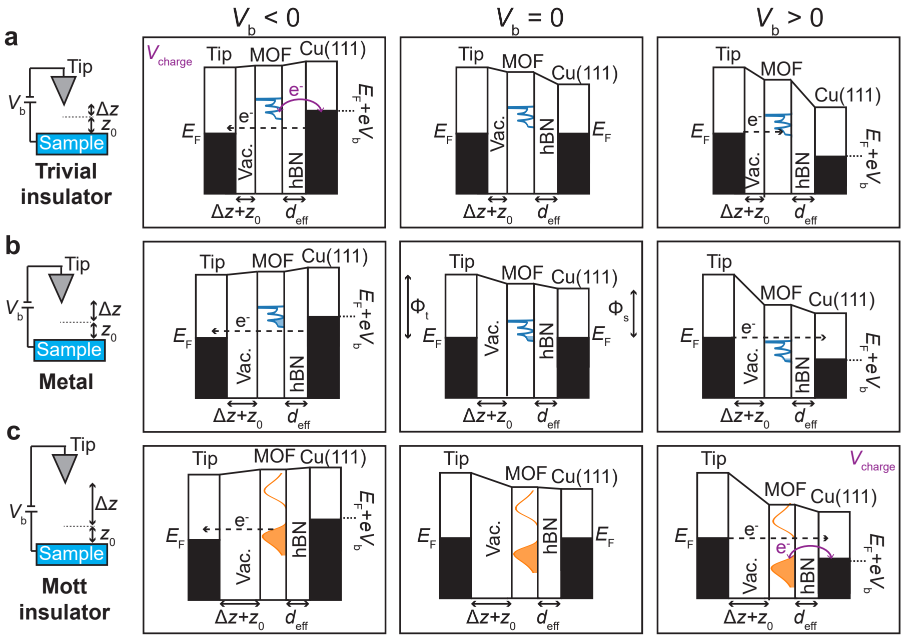

Supplementary Fig. 26: **Double-barrier tunnelling junction (DBTJ) model of tip-induced gating of MOF at wire region of hBN/Cu(111) moiré pattern. a-c,** Schematics and energy diagrams of tip/vacuum/-MOF/hBN/Cu(111) DBTJ, for small, intermediate and large tip-sample distances $\Delta z + z_0$. When V_b is applied to the Cu(111) substrate (with tip grounded), a voltage drop occurs at the MOF location, resulting in MOF energy level shifts. These energy shifts can lead to MOF charging and to effective emptying/filling of MOF electronic states, allowing for gating of MOF transitions, from (correlated) Mott insulator to metal to trivial insulator. Some of these panels are shown in Fig. 4d of the main text. These schematics are qualitative representations of the proposed mechanism of tip-induced gating.

Here, we provide more detailed information on this tip-induced transition from Mott insulator to metal to trivial insulator, and on the charging of MOF electronic states.

The dI/dV for the MOF at the hBN/Cu(111) moiré wire region in Fig. 4d of the main text show an electronic gap, with a clear peak (sharper than the near-Fermi band features in Fig. 3b, with a maximum indicated by purple circles) at positive bias voltage for large tip-sample distances, and at negative bias voltage for small tip-sample distances (see also Supplementary Fig. 28). These spectra also show a subtler band edge (indicated by the red squares in main text Fig. 4d, similar to the near-Fermi band features in Fig. 3b) at a bias voltage of sign opposite to that of the sharp peak – at negative bias voltage for large tip-sample distances and at positive bias voltage for small

tip-sample distances. For intermediate tip-sample distances, these spectra are gapless, with a significantly larger Fermi-level dI/dV signal (see main text Fig. 4f). From these observations, we infer that the MOF is an insulator (trivial or Mott) for large and small tip-sample distances (dI/dV spectra with a gap at the Fermi level), and a metal for intermediate tip-sample distances (dI/dV spectra with no gap at the Fermi level). The bias voltage position of the sharp peak (purple circles in Fig. 4d) increases linearly with respect to tip-sample distance, whereas the bias voltage position of the subtler band edge decreases non-linearly with tip-sample distance. Notably, DBTJ model Eqs. (1) and (2) in the main text provide very good fits for the bias voltage positions of both sharp peak and subtle band edge as a function of tip-sample distance (black curves in main text Fig. 4e). This provides compelling evidence that the sharp peaks (purple circles) are associated with charging of an intrinsic MOF electronic state lying at the edge (red squares) of a populated (large tip-sample distances) or empty (small tip-sample distances) band (see main text Fig. 4a-c and Supplementary Fig. 26).

In Fig. 2 of the main text, we show that the dI/dV spectra for the MOF at a pore region of the hBN/Cu(111) moiré pattern show a $\sim$200 meV electronic energy gap at the Fermi level. These spectra resemble the spectral function of the 2D kagome MOF in the Mott insulating phase (main text Fig. 1e), calculated via DMFT with a chemical potential that is consistent with the DFT-predicted occupation of the near-Fermi kagome bands for the MOF on hBN/Cu(111) (main text Fig. 1d, Supplementary Fig. 2). At an adjacent moiré wire region, the local work function increases by $\sim$0.2 eV for the specific periodicity of the MOF/hBN/Cu(111) domain considered in main text Fig. 3. Accordingly, the near-Fermi electronic states of the MOF at this moiré wire region are shifted upwards in energy in comparison to the near-Fermi electronic states of the MOF at the moiré pore region. The dI/dV spectra in Fig. 3b for this moiré wire region (for the specific tunnelling parameters used) show no gap at the Fermi level, with a significant non-zero Fermi-level dI/dV signal (main text Fig. 3e), indicative of a metallic phase. These experimental dI/dV spectra are consistent with the spectral function $A(E)$ of the MOF calculated via DMFT for a chemical potential that is reduced (in comparison with the DMFT calculations for the moiré pore region). This DMFT spectral function exhibits a significant magnitude and no gap at the Fermi level, indicating a metallic phase, resulting from the depopulation of the MOF bands (main text Fig. 3d).

From these observations we infer that: (i) the MOF at the moiré pore region is in a Mott insulating phase, and (ii) the MOF at the adjacent moiré wire region, for the specific tunnelling parameters used (bias voltage, tip-sample distance) in main text Fig. 3b, is in a metallic phase (as the result of the depopulation of the MOF near-Fermi electronic states due to the increase in local work function).

The work function of the STM tip is larger than that of the sample at the moiré wire region (Supplementary Note 18). When the tip-sample distance is reduced, the DBTJ leads to an upward energy shift of the MOF electronic states (with respect to the Cu(111) Fermi level): the MOF electronic states become further depopulated (Supplementary Fig. 26). That is, when the dI/dV spectra in main text Fig. 4d transition from gapless (metallic) to gapped (insulator) as the tip-sample distance decreases, we infer that the MOF electronic states associated with the MOF kagome bands become empty, with the Fermi level lying below the bottom of the three near-Fermi kagome bands (Supplementary Fig. 1). From this, we associate the gapped dI/d spectra at the top of main text Fig. 4d (for small tip-sample distances) to a trivial insulating phase of the MOF.

By contrast, when the tip-sample distance is increased (large Δz regime in Fig. 4 of main text), the DBTJ leads to a downward energy shift of MOF electronic states [with respect to the Cu(111) Fermi level], which become more populated (Supplementary Fig. 26). When the dI/dV spectra in main text Fig. 4d transition from gapless (metallic) to gapped (insulator) as the tip-sample distance increases, we infer that the population of the MOF electronic states associated with the MOF kagome bands also increases in turn, reaching a threshold (half-filling of the bands) that opens the Mott gap, with the Fermi lying in such Mott gap. This population of MOF states (here driven by the DBTJ effect and the increase in tip-sample distance) is analogous to the population of MOF states at the adjacent moiré pore region (due to the smaller local work function at such a moiré pore region; main text Fig. 3c). From this, we associate the gapped dI/dV spectra at the bottom of main text Fig. 4d (for large tip-sample distances) to the Mott insulating phase of the MOF.

This inference of a Mott gap at the moiré wire region for large tip-sample distances is supported by DMFT. Indeed, when the chemical potentials used in the DMFT calculations of main text Fig. 3d are all offset upwards by 45 meV (leading to further population of the MOF electronic states, mimicking the effect of a tip-sample distance increase), all spectra lie in the Mott insulating regime with no evidence of metallic character (Supplementary Note 4).

Supplementary Note 20 Tip-induced gating at moiré pore region

In addition to the tip-sample distance dependent dI/dV spectra shown in Fig. 4 of the main text for the wire region of the hBN/Cu(111) moiré pattern, we performed equivalent measurements at the pore region. These are shown in Supplementary Fig. 27. All these spectra exhibit an energy gap for all values of tip-sample distance $\Delta z + z_0$ considered, with no indication of tip-induced MOF charging or of Mott metal-insulator transition. We infer that, at this pore site, the MOF remains robustly within the Mott insulating phase for all tip-sample distances explored.

These dI/dV spectra do show however small variations of the lower Hubbard band maximum (LHBM), V_{LHB}, and upper Hubbard band minimum (UHBM), V_{UHB}, as a function of Δz (Supplementary Fig. 27b). According to the DBTJ model [see Eq. (1) in the main text], this variation should follow:

$$V_{\mathrm{LHB}}(\Delta z) = \frac{d_{\mathrm{eff}}\left(V_{\infty}^{(\mathrm{LHB})} + \Delta\Phi_{\mathrm{ts}}\right)}{(\Delta z + z_0)} + V_{\infty}^{(\mathrm{LHB})}, \tag{7}$$

$$V_{\mathrm{UHB}}(\Delta z) = \frac{d_{\mathrm{eff}}\left(V_{\infty}^{(\mathrm{UHB})} + \Delta\Phi_{\mathrm{ts}}\right)}{(\Delta z + z_0)} + V_{\infty}^{(\mathrm{UHB})}, \tag{8}$$

where d_{eff} is the effective width of the hBN tunnel barrier, $\Delta z + z_0$ is the width of the vacuum tunnel barrier (tip-MOF distance, with z_0 representing an initial tip-sample distance with respect to an STM setpoint), $V_{\infty}^{(\mathrm{LHB})}$ ($V_{\infty}^{(\mathrm{UHB})}$) is the bias voltage corresponding to the LHBM (UHBM, respectively) as $\Delta z \to \infty$, and $\Delta\Phi_{\mathrm{ts}}$ is the difference between tip and sample work functions [15].

We fit $V_{\mathrm{LHB}}(\Delta z)$ and $V_{\mathrm{UHB}}(\Delta z)$ according to Supplementary Eqs. (7), (8), by using a global fitting approach which maintained the same values of d_{eff}, z_0 and $\Delta\Phi_{\mathrm{ts}}$ for both fits (dashed black lines in Supplementary Fig. 27b). These fits show good agreement between the experimental data and the DBTJ model.

The change in energy of the LHBM and UHBM with Δz is more gradual at the pore site than the LHBM at the wire site (see Fig. 4 in the main text). While the difference between tip and sample work functions is expected to be larger at the pore site than at the wire (see Supplementary Note 18), this may be explained by a smaller d_{eff} at the pore site than at the wire site. For our system, $d_{\mathrm{eff}} = d_{\mathrm{hBN}}/\epsilon$, where d_{hBN} is the MOF-Cu(111) distance (∼hBN thickness) and ϵ is the out-of-plane relative dielectic constant associated with the hBN tunnel barrier [15]. From the fits in Supplementary Fig. 27c and Fig. 4e of the main text, we find $d_{\mathrm{eff}}^{(\mathrm{pore})} = 0.06 \pm 0.05$ Å and $d_{\mathrm{eff}}^{(\mathrm{wire})} = 0.2 \pm 0.1$ Å. While the hBN/Cu(111) moiré pattern is predominantly an electronic effect, some estimates of the structural corrugation between pore and wire sites are as large as 0.7 Å [19] – consistent with our findings for d_{eff}. This suggests that within the DBTJ model, the MOF at the wire regions of the hBN/Cu(111) moiré pattern may be more decoupled from the underlying Cu(111) than the MOF at the pore regions.

Additionally, no evidence of charging features were observed within the measurements acquired for the MOF at the pore sites of the hBN/Cu(111) moiré pattern. This can be explained by a larger energy difference between LHBM and E_{F} at the pore regions compared to the wire regions, meaning that greater values of V_{b} are required to induce charging.

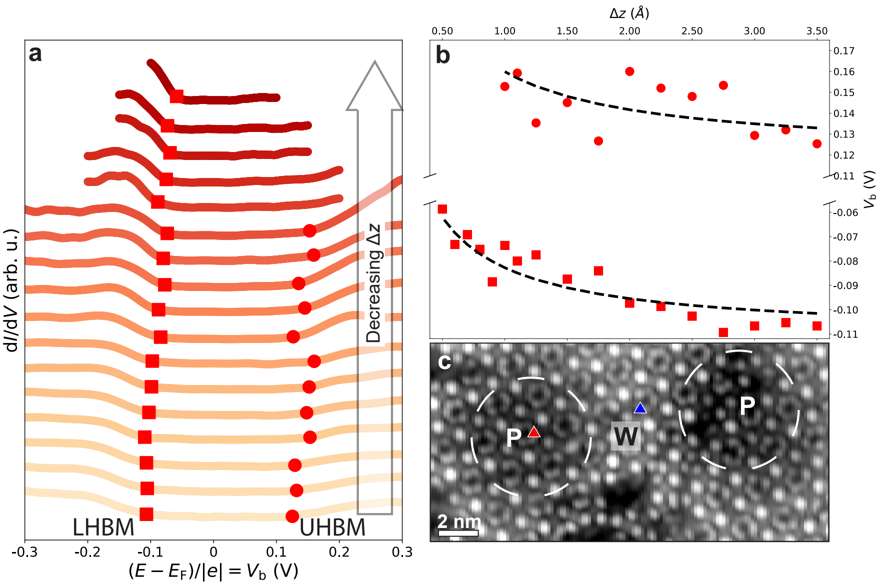

Supplementary Fig. 27: **dI/dV spectroscopy for DCA_3Cu_2 MOF at the centre of a pore region of hBN/Cu(111) moiré pattern**. **a,** dI/dV spectra for different tip-sample distances $\Delta z + z_0$ (z_0 represents tip-sample distance for STM setpoint of $V_b = 10$ mV, $I_t = 10$ pA) at a DCA lobe site (location indicated by red triangle in c). Red squares (circles) indicate LHBM (UHBM). **b,** LHBM and UHBM as a function of Δz, from (a). Black dashed curves: fits based on Supplementary Eqs. (7) and (8). **c,** STM image showing DCA_3Cu_2 MOF at pore and wire regions of hBN/Cu(111) moiré pattern. Red triangle indicates position where spectra shown in (a) were acquired; blue triangle indicates position where spectra shown in Fig. 4 of main text were acquired ($V_b = -1$ V, $I_t = 10$ pA).

Supplementary Note 21 Tip-induced gating at Cu sites; DBTJ model fit parameters

In Fig. 4 and Supplementary Fig. 27, we showed tip-induced gating of the MOF at a DCA lobe site located at a wire and at a pore region of the hBN/Cu(111) moiré pattern (with moiré period $\lambda \approx 12.5$ nm), respectively. Here, we demonstrate that measurements performed at MOF Cu sites are similar and consistent (Supplementary Figs. 28, 29).

Supplementary Fig. 28 shows dI/dV spectra for different tip-sample distances $z_0 + \Delta z$, performed on a Cu site of the DCA_3Cu_2 MOF at a wire region of the hBN/Cu(111) moiré pattern (with moiré periodicity $\lambda \approx 12.5$ nm, as in the main text). As in Fig. 4, the spectra acquired at this site feature, for large Δz, at negative V_b, a LHB whose energy maximum (LBHM)

increases (approaches the Fermi level) with decreasing Δz. These spectra also exhibit, at positive V_{b}, a peak whose energy position decreases linearly (and more abruptly than the LHBM) with decreasing Δz. We tracked the LHBM (assigned using the method outlined in Supplementary Note 13) and the positive bias peak (assigned as a local maximum in $\mathrm{d}I/\mathrm{d}V$) as a function of Δz, and then fit both features using Eqs. (1) and (2) of the main text, respectively, via a global fitting approach (see also main text Methods section 'DBTJ model'). The strong agreement between fits and data in Supplementary Fig. 28b shows that the DBTJ model captures the experimental behaviour well, with the positive bias peak corresponding to the charging of electronic states at the LHBM, as for Fig. 4. Similar to Fig. 4, as Δz is further decreased (for $\Delta z < 1.2$ Å), we observe a significant increase in Fermi-level ($V_{\mathrm{b}} = 0$) $\mathrm{d}I/\mathrm{d}V$ signal. As for the DCA lobe site in Fig. 4 of the main text, we interpret this as the transition from Mott insulator to metal via tip-induced gating (combined with the influence of the local work function at this moiré wire region). Within the range of experimental variables explored, the transition to a trivial insulator (see top curves in Fig. 4d of main paper) was not reached for this particular Cu site dataset.

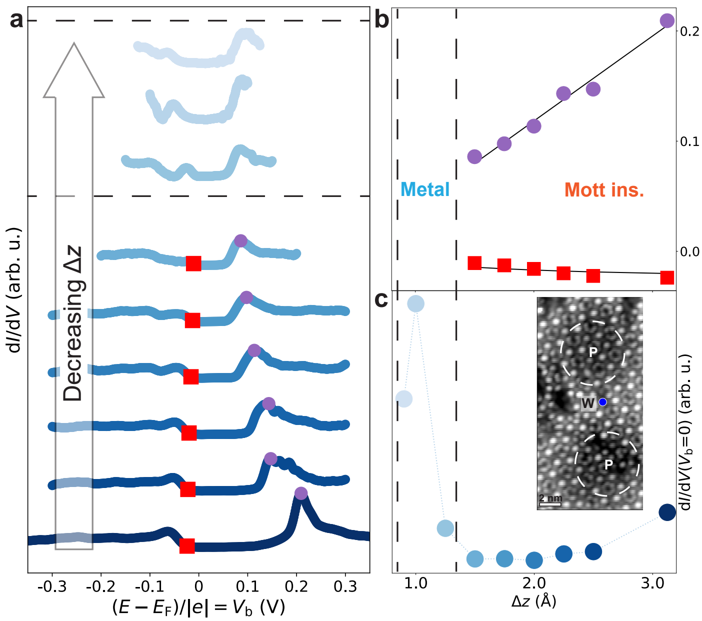

Supplementary Fig. 28: **Tip-induced gating at a Cu site of the MOF within a wire region of the hBN/Cu(111) moiré pattern. a,** $\mathrm{d}I/\mathrm{d}V$ spectra at MOF Cu site, for different $\Delta z + z_0$ (z_0 given by STM setpoint $V_\mathrm{b} = 10$ mV, $I_\mathrm{t} = 10$ pA). Purple circles (red squares): MOF charging peak (intrinsic electronic state at MOF band edge, respectively). Spectra normalised and offset for clarity. **b,** V_charge [purple circles in (a)] and V_state [red squares in (a)] as a function of Δz. Black solid lines: global fits to Eqs. (1) and (2) in main text. **c,** $\mathrm{d}I/\mathrm{d}V$ signal at Fermi level ($V_\mathrm{b} = 0$) as a function of Δz, from (a). Increased $\mathrm{d}I/\mathrm{d}V(V_\mathrm{b} = 0)$ indicates metallic phase. Inset: STM image with position where $\mathrm{d}I/\mathrm{d}V(\Delta z)$ were performed indicated by blue circle ($V_\mathrm{b} = -1$ V, $I_\mathrm{t} = 10$ pA).

Similarly, Supplementary Fig. 29a shows $\mathrm{d}I/\mathrm{d}V$ spectra for different tip-sample distances $z_0 + \Delta z$, performed on a Cu site of the DCA_3Cu_2 MOF at a pore region of the hBN/Cu(111) moiré pattern (with moiré periodicity $\lambda \approx 12.5$ nm, as in the main text). Consistent with Supplementary Fig. 27 for measurements at a DCA lobe site, Supplementary Fig. 29b shows the evolution of LHBM and UHBM as a function of Δz, well described by the DBTJ model Supplementary Eqs. (7) and (8) (dashed curves), respectively.

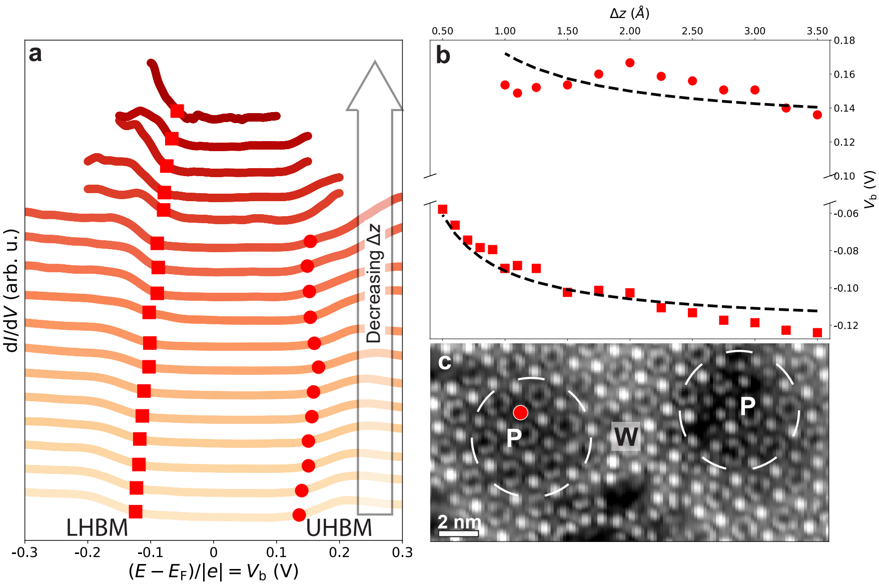

Supplementary Fig. 29: **dI/dV spectroscopy for Cu site of MOF within a pore region of hBN/Cu(111) moiré pattern**. **a,** dI/dV spectra for different tip-sample distances $\Delta z + z_0$ (z_0 represents tip-sample distance for STM setpoint of $V_b = 10$ mV, $I_t = 10$ pA) at a Cu site [location indicated by red circle in (c)]. Red squares (circles) indicate LHBM (UHBM, respectively). **b,** LHBM and UHBM as a function of Δz, from (a). Black dashed curves: fits based on Supplementary Eqs. (7) and (8). **c,** STM image of DCA_3Cu_2 MOF on hBN/Cu(111). Red circle indicates position where spectra shown in (a) were acquired ($V_b = -1$ V, $I_t = 10$ pA).

The fitting parameters corresponding to the fits in Fig. 4e (main text), and Supplementary Figs. 27b, 28b, and 29b, are summarised in Supplementary Table 1. Equations (1) and (2) of the main text were used to fit the spectra acquired at wire regions of the hBN/Cu(111) moiré pattern in Fig. 4e of the main text (bottom curves) and Supplementary Fig. 28b (and exhibiting a gap at the Fermi level), with fitting parameters $V_\infty^{(\mathrm{Mott})}$, d_{eff}, $\Delta\Phi_{\mathrm{ts}}$, and z_0. For the top spectra in Fig. 4e of the main text (that we attribute to the trivial insulator phase), we used the same two equations with fitting parameter $V_\infty^{(\mathrm{kagome})}$ corresponding to the energy level at the bottom of the near-Fermi kagome bands. Supplementary Eqs. (7) and (8) were used to fit spectra acquired at the pore regions of the hBN/Cu(111) moiré pattern in Supplementary Figs. 27 and 29 with fitting parameters $V_\infty^{(\mathrm{LHB})}$, $V_\infty^{(\mathrm{UHB})}$, d_{eff}, $\Delta\Phi_{\mathrm{ts}}$, and z_0.

The fitting parameters $V_\infty^{(\mathrm{Mott/LHB})}$, $V_\infty^{(\mathrm{UHB})}$ and d_{eff} – characteristic of the MOF system on hBN/Cu(111) – obtained for the fit curves in Fig. 4e of the main text, and Supplementary Figs. 27b, 28b for DCA lobe and Cu

sites are highly consistent (within uncertainty), for both pore and wire regions of the hBN/Cu(111) moiré pattern. The global fitting approach used does lead to large uncertainties (determined from fitting procedure as one standard deviation of fit parameters) in some cases, however, notably for values of z_0. As discussed in the previous Supplementary Note 20, the difference in d_{eff} between pore and wire regions is highly consistent with hBN/Cu(111) literature and supports our claim that the MOF is slightly more decoupled at the wire regions than at the pore regions [19]. Importantly, for data acquired with the same tip (DCA lobe measurements at both pore and wire regions, and Cu measurements at the pore region), $\Delta\Phi_{\mathrm{ts}}^{(\mathrm{pore})} - \Delta\Phi_{\mathrm{ts}}^{(\mathrm{wire})} = (\Phi_{\mathrm{t}} - \Phi_{\mathrm{pore}}) - (\Phi_{\mathrm{t}} - \Phi_{\mathrm{wire}}) = \Phi_{\mathrm{wire}} - \Phi_{\mathrm{pore}} \approx 0.2$ eV, consistent with the expected local work function corrugation of the hBN/Cu(111) moiré pattern of interest with $\lambda \approx 12.5$ nm. Similarly, the difference in $V_{\infty}^{(\mathrm{Mott/LHB})}$ at pore and wire regions – with $V_{\infty}^{(\mathrm{Mott/LHB})}$ significantly closer to the Fermi level at the wire region than at the pore region – is consistent with the findings shown in Fig. 3 of the main text and Supplementary Figs. 19-22. Finally, it is important to note that the condition of the STM tip was different for measurements at the Cu site in a moiré wire region to the other three datasets, providing a potential explanation for the different values of $\Delta\Phi_{\mathrm{ts}}$ and z_0. Nonetheless, as for the other datasets, a $\Delta\Phi_{\mathrm{ts}} > 0$ was obtained from the fit at such wire region Cu site, indicating a tip-induced gating behaviour consistent with other measurements. This provides further evidence that the DBTJ model quantitatively captures the observed changes in $\mathrm{d}I/\mathrm{d}V$ measurements with changing tip-sample distance.

Quantity	DCA lobe sites		Cu sites	
	Pore	Wire	Pore	Wire*
$V_{\infty}^{(\mathrm{Mott/LHB})}$ (V)	-0.11 ± 0.01	-0.03 ± 0.02	-0.12 ± 0.02	-0.03 ± 0.02
$V_{\infty}^{(\mathrm{kagome})}$ (V)		-0.02 ± 0.01		
$V_{\infty}^{(\mathrm{UHB})}$ (V)	0.12 ± 0.01		0.13 ± 0.03	
d_{eff} (Å)	0.06 ± 0.05	0.2 ± 0.1	0.06 ± 0.08	0.4 ± 0.3
$\Delta\Phi_{\mathrm{ts}}$ (eV)	0.6 ± 0.4	0.36 ± 0.09	0.6 ± 0.5	0.1 ± 0.2
z_0 (Å)	0.2 ± 0.6	0.007 ± 0.5	0.03 ± 1	0.4 ± 2

Supplementary Table 1: DBTJ model fitting parameters. Eqs. (1) and (2) in main text were used to fit the $\mathrm{d}I/\mathrm{d}V$ spectra acquired at wire regions of the hBN/Cu(111) moiré pattern in Fig. 4e (main text) and Supplementary Fig. 28b. Supplementary Eqs. (7) and (8) were used to fit spectra acquired at the pore regions of the hBN/Cu(111) moiré pattern in Supplementary Figs. 27 and 29. *The Cu site dataset at the moiré wire region was acquired with a different STM tip to the other three datasets.

Supplementary Note 22 Tip-induced gating: evidence of metallic character of MOF at wire region

In Fig. 4 of the main text, we show that the MOF undergoes a transition from a Mott insulating phase to a metal phase to a trivial insulator phase via tip-induced gating at the wire region of the hBN/Cu(111) moiré pattern. The metallic phase is evidenced by the absence of an energy gap at the Fermi level, for a small window of intermediate tip-sample distances $\Delta z + z_0$. Notably, the $\mathrm{d}I/\mathrm{d}V$ spectra acquired at these distances have increased $\mathrm{d}I/\mathrm{d}V(V_\mathrm{b} = 0)$ signal; see Supplementary Fig. 30b-c. This is indicative of an increased spectral function at the Fermi level, and hence of metallic character.

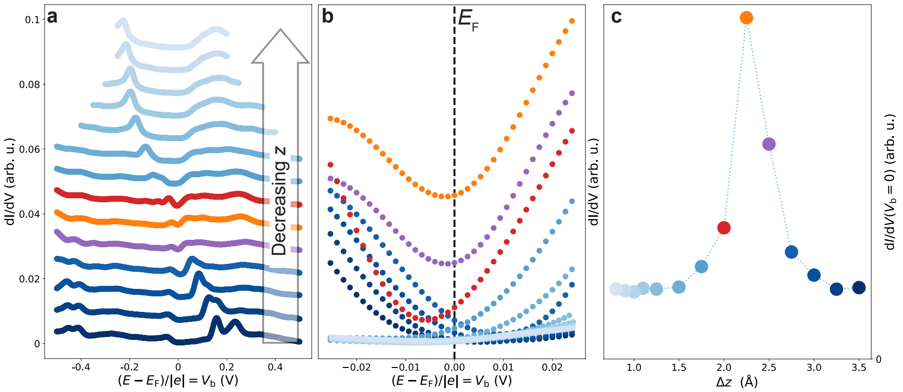

Supplementary Fig. 30: **Evidence of tip-induced metallic character of DCA_3Cu_2 MOF at wire region of hBN/Cu(111) moiré pattern. a,** Normalised $\mathrm{d}I/\mathrm{d}V(\Delta z)$ spectra (also shown in Fig. 4 of main text) acquired at DCA anthracene extremity site at wire region, for different tip-sample distances Δz ($\Delta z = 0$ corresponds to STM setpoint of $V_\mathrm{b} = 10$ mV, $I_\mathrm{t} = 10$ pA). Curves offset for clarity. **b,** Same $\mathrm{d}I/\mathrm{d}V(\Delta z)$ spectra as in (a), for near-Fermi energy range (no offset). Purple, orange and red spectra for intermediate $\Delta z + z_0$ show increased $\mathrm{d}I/\mathrm{d}V$ signal at Fermi level ($V_\mathrm{b} = 0$), suggesting metallic character with no gap at the Fermi level. Vertical black dashed line: Fermi level. **c,** Value of $\mathrm{d}I/\mathrm{d}V$ at E_F ($V_\mathrm{b} = 0$) as a function of Δz, illustrating metallic character for the purple, orange and red points.

Supplementary Note 23 Temperature-dependent measurements and calculations

To assess the influence of temperature on the electronic properties of the DCA_3Cu_2 MOF, we conducted further STS measurements at both pore and wire sites of the hBN/Cu(111) moiré pattern, at a temperature of 77 K (Supplementary Fig. 31a). Supplementary Fig. 31b shows measurements at 4 K, for comparison. While the 77 K spectra appear broader than the 4 K spectra (due to thermal broadening), they qualitatively show the same electronic features: metallic behaviour at the wire region (blue, with no gap at the Fermi level and a larger Fermi-level $\mathrm{d}I/\mathrm{d}V$ signal), and insulating behaviour at the pore region (orange, with an energy gap at the Fermi level).

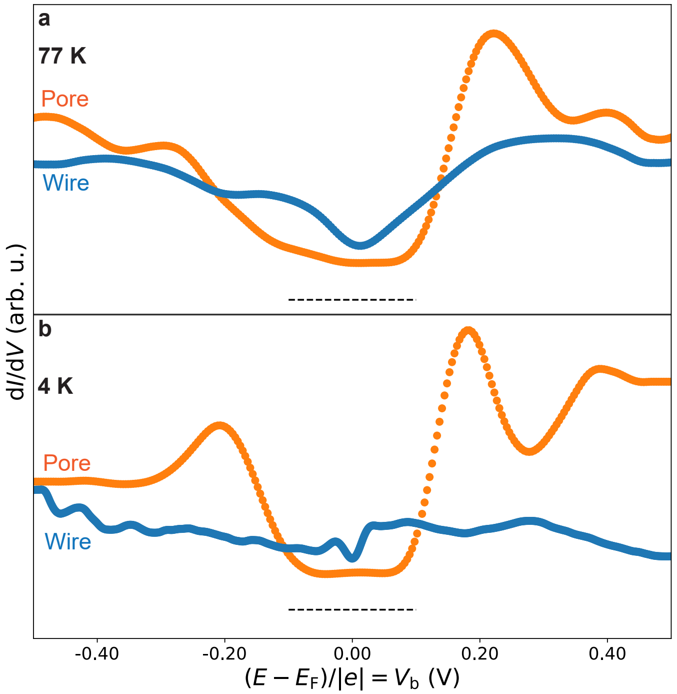

Supplementary Fig. 31: **Temperature-dependent STS measurements.** $\mathrm{d}I/\mathrm{d}V$ measurements performed at DCA lobe sites of the DCA_3Cu_2 MOF at hBN/Cu(111) moiré pore (orange) and wire (blue) regions, at temperatures of **(a)** 77 K and **(b)** 4 K. Setpoints: (a) $V_\mathrm{b} = -500$ mV, $I_\mathrm{t} = 100$ pA; (b) $V_\mathrm{b} = -500$ mV, $I_\mathrm{t} = 500$ pA (orange), and $V_\mathrm{b} = -500$ mV, $I_\mathrm{t} = 160$ pA (blue). Black dashed horizontal lines indicate position of $\mathrm{d}I/\mathrm{d}V = 0$.

We also explored temperature-dependence using DMFT. Supplementary Fig. 32 shows the evolution of DMFT spectral functions, $A(E)$, for different temperatures, with $U = 0.65$ eV and a chemical potential E_F corresponding to the pore (Supplementary Fig. 32a) or wire regions (Supplementary Fig. 32b) of the hBN/Cu(111) moiré pattern. DMFT predicts no qualitative change in the MOF's electronic properties as the temperature evolves from 29 K to beyond room temperature, consistent with the experimental observations in Supplementary Fig. 31.

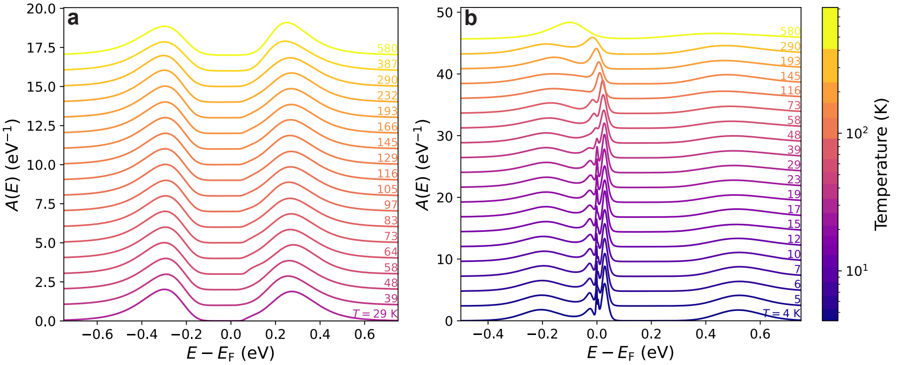

Supplementary Fig. 32: **Temperature-dependent spectral functions calculated by DMFT. a,** $E_F = 0.4$ eV (∼half-filling, corresponding to moiré pore regions). **b,** $E_F = 0.2$ eV (filling corresponding to metal-like phase observed experimentally at wire regions). $U = 0.65$ eV.

Supplementary Note 24 STS measurements at different moiré pore regions

To examine how consistent the electronic properties of the DCA_3Cu_2 MOF are across the surface, we acquired dI/dV spectra at Cu and DCA lobe MOF sites, at different moiré pore regions within a hBN/Cu(111) domain with moiré period $\lambda \approx 12.5$ nm (Supplementary Fig. 33). These spectra are very similar to each other, showing LHB and UHB signatures, and a gap $E_g \approx 0.2$ eV at the Fermi level. Some subtle differences (e.g., shifting in energy of Hubbard bands) may be attributed to spectra acquired at slightly different distances from the moiré pore centre (hence with a slightly different hBN/Cu(111) local work function), or to different setpoints (see Supplementary Note 20). This shows the consistency of the MOF electronic properties at equivalent sites within a hBN/Cu(111) domain.

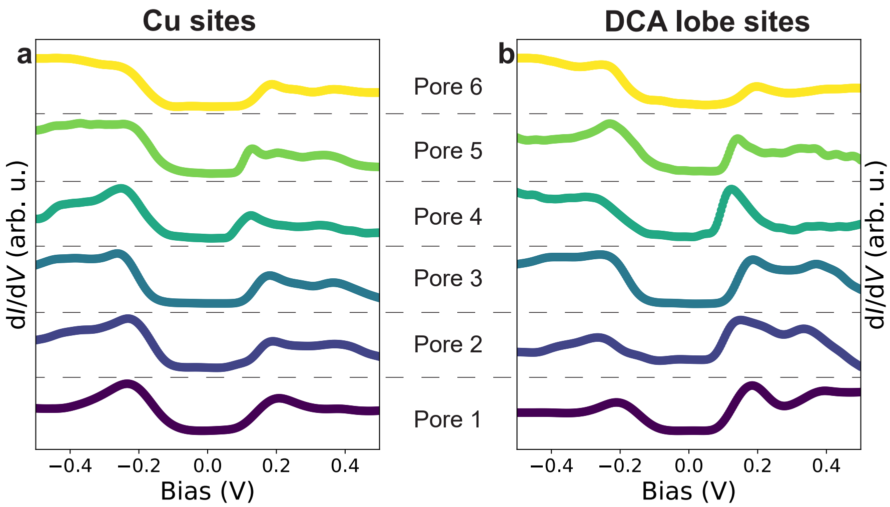

Supplementary Fig. 33: **dI/dV spectra at different moiré pore regions within hBN/Cu(111) domain with moiré period $\lambda \approx$ 12.5 nm.** **a,** Cu MOF sites. **b,** DCA lobe sites. Acquisition sites close to centre of moiré pore regions. Spectra normalised and offset for clarity. Pore 1 setpoint: $V_\mathrm{b} = -500$ mV, $I_\mathrm{t} = 500$ pA. Pores 2,3 setpoints: 190 pm further from surface with respect to setpoint of $V_\mathrm{b} = 10$ mV, $I_\mathrm{t} = 10$ pA. Pores 4,5 setpoint: 225 pm further from surface with respect to setpoint of $V_\mathrm{b} = 10$ mV, $I_\mathrm{t} = 10$ pA. Pore 6 setpoint: 150 pm further from surface with respect to setpoint of $V_\mathrm{b} = 10$ mV, $I_\mathrm{t} = 10$ pA.

References

[1] Zhang, L. Z. *et al.* Intrinsic Two-Dimensional Organic Topological Insulators in Metal–Dicyanoanthracene Lattices. *Nano Lett.* **16** (3), 2072–2075 (2016) .

[2] Silver, R. N., Sivia, D. S. & Gubernatis, J. E. Maximum-entropy method for analytic continuation of quantum Monte Carlo data. *Phys. Rev. B* **41** (4), 2380–2389 (1990) .

[3] Zhang, Q. *et al.* Tuning Band Gap and Work Function Modulations in Monolayer hBN/Cu(111) Heterostructures with Moiré Patterns. *ACS Nano* **12** (9), 9355–9362 (2018) .

[4] Georges, A., Kotliar, G., Krauth, W. & Rozenberg, M. J. Dynamical mean-field theory of strongly correlated fermion systems and the limit of infinite dimensions. *Rev. Mod. Phys.* **68** (1), 13–125 (1996) .

[5] Mahan, G. D. *Many-Particle Physics* 3 edn. Physics of Solids and Liquids (Kluwer Academic/Plenum, New York, NY, 2000).

[6] Field, B., Schiffrin, A. & Medhekar, N. V. Correlation-induced magnetism in substrate-supported 2D metal-organic frameworks. *npj Comput Mater* **8** (1), 1–10 (2022) .

[7] Skornyakov, S. L., Skorikov, N. A., Lukoyanov, A. V., Shorikov, A. O. & Anisimov, V. I. LDA + DMFT spectral functions and effective electron mass enhancement in the superconductor LaFePO. *Phys. Rev. B* **81** (17), 174522 (2010) .

[8] Adler, R., Kang, C.-J., Yee, C.-H. & Kotliar, G. Correlated materials design: prospects and challenges. *Rep. Prog. Phys.* **82** (1), 012504 (2018) .

[9] Kotliar, G. & Vollhardt, D. Strongly Correlated Materials: Insights From Dynamical Mean-Field Theory. *Physics Today* **57** (3), 53–59 (2004) .

[10] Vollhardt, D. Dynamical mean-field theory for correlated electrons. *Annalen der Physik* **524** (1), 1–19 (2012) .

[11] Kumar, D., Hellerstedt, J., Lowe, B. & Schiffrin, A. Mesoscopic 2D molecular self-assembly on an insulator. *Nanotechnology* **34** (20), 205601 (2023) .

[12] Yan, L. *et al.* Synthesis and Local Probe Gating of a Monolayer Metal-Organic Framework. *Advanced Functional Materials* **31** (22), 2100519 (2021) .

[13] Chen, Y. *et al.* Strong correlations and orbital texture in single-layer 1T-$TaSe_2$. *Nat. Phys.* **16** (2), 218–224 (2020) .

[14] Kumar, D. *et al.* Manifestation of Strongly Correlated Electrons in a 2D Kagome Metal–Organic Framework. *Advanced Functional Materials* **31** (48), 2106474 (2021) .

[15] Kumar, D., Krull, C., Yin, Y., Medhekar, N. V. & Schiffrin, A. Electric Field Control of Molecular Charge State in a Single-Component 2D Organic Nanoarray. *ACS Nano* **13** (10), 11882–11890 (2019) .

[16] Joshi, S. *et al.* Boron Nitride on Cu(111): An Electronically Corrugated Monolayer. *Nano Lett.* **12** (11), 5821–5828 (2012) .

[17] Joshi, S. *et al.* Control of Molecular Organization and Energy Level Alignment by an Electronically Nanopatterned Boron Nitride Template. *ACS Nano* **8** (1), 430–442 (2014) .

[18] Auwärter, W. Hexagonal boron nitride monolayers on metal supports: Versatile templates for atoms, molecules and nanostructures. *Surface Science Reports* **74** (1), 1–95 (2019) .

[19] Schwarz, M. *et al.* Corrugation in the Weakly Interacting Hexagonal-BN/Cu(111) System: Structure Determination by Combining Noncontact Atomic Force Microscopy and X-ray Standing Waves. *ACS Nano* **11** (9), 9151–9161 (2017) .
